# Towards a Canary Islands barcode database for soil biodiversity: revealing cryptic and unrecorded mite species diversity within insular soils

**DOI:** 10.3897/BDJ.12.e113301

**Published:** 2024-01-10

**Authors:** Irene Santos-Perdomo, Daniel Suárez, María L. Moraza, Paula Arribas, Carmelo Andújar

**Affiliations:** 1 Island Ecology and Evolution Research Group, Institute of Natural Products and Agrobiology (IPNA-CSIC), 38206, La Laguna, Spain Island Ecology and Evolution Research Group, Institute of Natural Products and Agrobiology (IPNA-CSIC), 38206 La Laguna Spain; 2 School of Doctoral and Postgraduate Studies, University of La Laguna, 38206, La Laguna, Spain School of Doctoral and Postgraduate Studies, University of La Laguna, 38206 La Laguna Spain; 3 Universidad de Navarra, Instituto de Biodiversidad y Medioambiente BIOMA, Irunlarrea 1, 31008, Pamplona, Spain Universidad de Navarra, Instituto de Biodiversidad y Medioambiente BIOMA, Irunlarrea 1, 31008 Pamplona Spain

**Keywords:** Acari, COI, barcoding, soil mesofauna, biotic frontier, species inventory, oceanic islands, species delimitation methods

## Abstract

Soil arthropod diversity contributes to a high proportion of the total biodiversity on Earth. However, most soil arthropods are still undescribed, hindering our understanding of soil functioning and global biodiversity estimations. Inventorying soil arthropods using conventional taxonomical approaches is particularly difficult and costly due to the great species richness, abundance and local-scale heterogeneity of mesofauna communities and the poor taxonomic background knowledge of most lineages. To alleviate this situation, we have designed and implemented a molecular barcoding framework adapted to soil fauna. This pipeline includes different steps, starting with a morphology-based selection of specimens which are imaged. Then, DNA is extracted non-destructively. Both images and voucher specimens are used to assign a taxonomic identification, based on morphology that is further checked for consistency with molecular information. Using this procedure, we studied 239 specimens of mites from the Canary Islands including representatives of Mesostigmata, Sarcoptiformes and Trombidiformes, of which we recovered barcode sequences for 168 specimens that were morphologically identified to 49 species, with nine specimens that could only be identified at the genus or family levels. Multiple species delimitation analyses were run to compare molecular delimitations with morphological identifications, including ASAP, mlPTP, BINs and 3% and 8% genetic distance thresholds. Additionally, a species-level search was carried out at the Biodiversity Databank of the Canary Islands (BIOTA) to evaluate the number of species in our dataset that were not previously recorded in the archipelago. In parallel, a sequence-level search of our sequences was performed against BOLD Systems. Our results reveal that multiple morphologically identified species correspond to different molecular lineages, which points to significant levels of unknown cryptic diversity within the archipelago. In addition, we evidenced that multiple species in our dataset constituted new records for the Canary Islands fauna and that the information for these lineages within online genetic repositories is very incomplete. Our study represents the first systematic effort to catalogue the soil arthropod mesofauna of the Canary Islands and establishes the basis for the Canary Islands Soil Biodiversity barcode database. This resource will constitute a step forward in the knowledge of these arthropods in a region of special interest.

## Introduction

Soils harbour a vast proportion of total biodiversity on Earth (Decaëns et al. 2006), which play a crucial role in critical processes, such as soil formation, nutrient and water cycling, climate regulation, production of food, medicine and fibre, disease and pest control (Bardgett and Van Der Putten 2014). At the same time, the soil environment is considered one of the last biotic frontiers to human knowledge (André et al. 1994). Current knowledge gaps regarding soil biodiversity are still massive (Guerra et al. 2020), primarily because of the methodological and logistical complexity of approaching complex soil communities. A striking and concerning consequence of these knowledge gaps is the difficulty in developing and implementing conservation strategies to preserve soil biodiversity (Veresoglou et al. 2015), a direct impediment to the 2030 UN Agenda for Sustainable Development. Recently, the Convention on Biological Diversity and the FAO-Global Soil Partnership actively promoted diverse initiatives to fill critical knowledge gaps for soil biodiversity, starting with the first report on the State of Knowledge of Soil Biodiversity (FAO et al. 2020).

The methodological and logistical issues that have hindered our understanding of soil biodiversity are particularly exacerbated for some edaphic groups and geographical areas (Guerra et al. 2020). A substantial fraction of soil biodiversity is represented by the soil mesofauna, i.e. small-bodied invertebrates measuring between 0.1 and 2 mm that are regularly found in their thousands in every square metre of soil (Walter and Proctor 2013, Nielsen et al. 2015). Soil arthropod mesofauna is a functionally important component of soil communities, directly affecting the physicochemical and biological properties of the soil and leaf litter (Ponge 2013). However, identifying and quantifying soil arthropod mesofauna using conventional taxonomical approaches is difficult and costly. This is due to the great species richness, abundance and local-scale heterogeneity of mesofauna communities, the minute size of specimens, the scarcity of taxonomic experts and the poor taxonomic background knowledge of most soil lineages (Bardgett 2002, Decaëns 2010). Besides, soil arthropod diversity estimations using morphological techniques could be substantially biased, at least in some lineages, due to the high prevalence of cryptic diversity within morphologically defined species (e.g. Cicconardi et al. (2013), Pérez-Delgado et al. (2022), Yin et al. (2022)). This situation could result in important species richness sub-estimations within soil arthropod fauna, a substantially misrepresented component of terrestrial animal biodiversity.

DNA barcoding, i.e. the use of short, standardised genomic regions to facilitate species identification and discovery, has revolutionised the study of biodiversity. Barcoding specimens using the standard barcode region (COI‐bcr) for metazoan DNA taxonomy (Folmer et al. 1994, Hebert et al. 2003a, Hebert et al. 2003b) can enable the assessment of soil mesofauna diversity in multiple ways. COI-bcr allows the delineation of molecular species, enabling the identification for many animal lineages within morphospecies and the detection of undescribed species (e.g. Janzen et al. (2017), Young et al. (2019)). Similarly, barcoded sequences, through their comparison with available COI‐bcr reference databases (e.g. International Barcode of Life Project, iBOL; http://www.ibol.org), provide a global context for species delimitation and identification (e.g. Ashfaq et al. (2017), Cicconardi et al. (2017)). The link between the morphology and the barcode sequences of specimens can be maintained via photographic records or non-destructive DNA extractions and so also provides a fundamental tool for the development of taxonomical knowledge in complex lineages (e.g. Chan et al. (2014), Jackson et al. (2014), Marconi et al. (2022)). Finally, generating COI‐bcr databases for specific areas provides a key resource for further implementation of biomonitoring using HTS tools, such as metabarcoding of soil samples (Andújar et al. 2018, Arribas et al. 2019, Andújar et al. 2022). The development of local barcode databases for the biotas of specific countries or regions was started more than a decade ago by pioneering projects (e.g. German Barcode of Life or the Swedish Malaise Trap Project) and it is revealed as a crucial step forward for biodiversity inventory and conservation in those areas (e.g. Hendrich et al. (2015), Young et al. (2019), Ronquist et al. (2020)). Extending these initiatives to understudied biodiversity fractions and hyperdiverse (and threatened) geographical regions is fundamental to the ongoing decline in biodiversity.

The Canary Islands are an oceanic archipelago within the subtropical region of the North Atlantic Ocean with great conservational and patrimonial value in both a national and European context. The Canary Islands are recognised as a Special Territory of the European Union, where quantifying and controlling biodiversity loss is a priority. The diversity of soil mesofauna within oceanic islands needs to be better explored. Literature on the topic is limited (but see Koh et al. (2002), Maraun et al. (2007), Fattorini (2009), Cicconardi et al. (2017)) and even basic species inventory data are, in general, scarce. Within the Canary Islands, the Biodiversity Databank of the Canary Islands (https://www.biodiversidadcanarias.es/biota/; hereafter referred as BIOTA) is a constantly updated public database containing all species records for the archipelago published in the scientific literature. BIOTA currently reports 474 species of Acari from the Canary Islands, with 288 species from the Island of Tenerife. Recently, a study implementing metabarcoding for a non-exhaustive set of soil samples from Tenerife has revealed nearly double the OTUs of Acari on the Island (Andújar et al. 2022). Remarkably, for most of the lineages (8% similarity clusters from COI metabarcoding data) inventoried in that study, the species-level molecular taxonomic identification was impossible due to the absence of reference barcode sequences.

Here, we initiate the Barcode Database of soil mesofauna from the Canary Islands (CISoilBiota) by: i) developing a standardised workflow that combines traditional morphological identification and COI barcoding of soil arthropod specimens within the framework of the BOLD System (Ratnasingham and Hebert 2007) and ii) by providing the first 168 barcodes from mite specimens collected in soils of the islands of Tenerife and Fuerteventura. This dataset was subsequently analysed as proof of concept to demonstrate the remarkable unrecorded mesofauna diversity present in the soils of the archipelago. Our study aims to provide the basis for the Canary Islands Soil Biodiversity barcode database (CISoilBiota), highlighting its strong potential for the biodiversity inventory and conservation of the soils of the Canary Islands.

## Material and methods

### Fieldwork and sample processing

Mite specimens were retrieved from 24 soil samples collected between 2018 and 2020 in different localities of laurel forests, pine forests, heathlands and crops of the islands of Tenerife and Fuerteventura (Suppl. material 1). At each site, superficial and deep soil samples were taken. The superficial sample included the litter and the first centimetres of the soil and the deep sample included 25 litres of soil from a hole about 30-40 cm in depth. Every sample was processed following the flotation-Berlese-flotation protocol (FBF) that allows for the ‘clean’ extraction of arthropod mesofauna (Arribas et al. 2016). Briefly, the FBF protocol is based on soil flotation in water, which allows the extraction of the organic matter and soil mesofauna from raw soil samples. Subsequently, the organic portion is placed in a modified Berlese apparatus to capture specimens alive and preserve them in absolute ethanol at -20ºC. The last step of the FBF protocol is an additional flotation of the ethanol-preserved arthropods, resulting in ‘clean’ bulk specimen samples. From the 24 cleaned samples of bulk mesofauna, which were examined under a Motic SMZ-171 stereoscope, 239 mites were selected, with representatives from the orders Mesostigmata, Sarcoptiformes and Trombidiformes and maximising the morphological variability of the subset. Each specimen was assigned a unique identifier number (voucher number) and henceforth is referred to as ‘vouchers’.

#### Photo recording and morphological identification

Before DNA extraction, several photos were taken of each intact voucher. A Canon EOS 750D camera attached to a microscope (Zeizz Axioskop 40) was used to take high-resolution pictures of the mite specimens submerged in ethanol and over a white background. For each voucher, a range from 30 to 90 photos was taken by using a 10x objective at different confocal distances depending on the size of each voucher, to subsequently compile with Zerene Stacker (Zerene Systems LLC) for a fully on-focus photographic record of each voucher. Photo alignment and stacking were done following PMax settings and the final image was saved in JPEG format with the highest quality (compression quality = 12). Images and mite vouchers, after non-destructive DNA extractions (see below), were subsequently studied by an expert taxonomist (co-author M.L. Moraza) for morphological identification. Each specimen was examined under a Nikon MSZ745 stereomicroscope and an Olympus Vanox with a phase contrast microscope. For further identification, specimens were cleared in Nesbitt’s liquid and mounted using Hoyer’s medium. Mite species were identified using taxonomic keys for the Palearctic Region (Gilyarov and Breguetova 1977, Gilyarov 1978, Karg 1993, Pérez-Íñigo 1993, Pérez-Íñigo 1997). Higher mite taxonomic categories follow Krantz and Walter (2009) classification.

### DNA extraction and sequencing

Non-destructive DNA extractions were performed for each voucher. For that purpose, the exoskeleton of each specimen was punctured with an entomological pin and kept individually in 1.5 ml vials. Pins were sterilised under a Bunsen burner to avoid contaminations between samples. A volume of 110 µl of digestion buffer (pK ratio 1/10) was added to each voucher and digestion was done overnight at 60°C. The supernatant (DNA lysate) was transferred to the corresponding well within a deep-well plate and the (DNA-extracted) vouchers were maintained in the vials with ethanol for further morphological study (see above). Subsequent steps of the DNA extractions used the MAg-bind Blood & Tissue DNA extraction Kit (Omega Bio-tek) in the KingFisher robotic system (Thermo Fisher Scientific inc.). The default protocol was followed, but DNA lysate and reagent volumes were cut in half. The resulting 100 µl of genomic DNA extraction were split into a ‘stock plate’ directly frozen at -20ºC and a ‘working plate’ that was used to quantify DNA concentration using absorbance values within an Infinite M Nano (Tecan Trading AG).

PCR amplification was done for the 5’ end COI gene (standard barcode region for Metazoa; Hebert et al. (2003b)) using degenerate Folmer barcode primers (Fol-degen-for: ‘TCNACNAAYCAYAARRAYATYGG’; Fol-degen-rev: ‘TANACYTCNGGRTGNCCRAARAAYCA’; Folmer et al. (1994), Yu et al. (2012)). For PCR reaction, 5 µl of extracted DNA was used with 15 µl of PCR mix, which consisted in: 9.72 µl molecular-grade water, 2 µl 10x NH_4_ buffer, 1.2 µl MgCl_2_, 0.4 µl dNTPs, 0.4 µl of BSA, 0.6 µl 10 µM Fol-degen-for and 0.6 µl 10 µM Fol-degen-rev primers and 0.08 µl Taq polymerase (BIOTAQ™ DNA Polymerase, Bioline) per sample. PCR conditions were: 10 min at 95°C in 10 min, followed by 44 cycles of 30 s at 95°C, 30 s at 48°C and 3 min at 72°C; 10 min at 72°C and holding at 10°C. After viewing PCR products in agarose gel 1%, the positive PCR products were purified. The cleaning mix was prepared with 0.005 µl of exonuclease (exo1), 0.050 µl of rapid alkaline phosphatase (rAP) and 1.945 µl of double distilled water. A volume of 2 µl per 5 µl of DNA sample was used to run a 30 min protocol in the thermal cycler (30 min at 37°C, 5 min at 95°C and holding at 12°C). After cleaning, 5 µl of Fol-degen-for 5 µM primer were added to each sample. The purified PCR product for each voucher was Sanger-sequenced with ABI technology in Macrogen, Spain.

### Sequence editing, submission and molecular phylogenetics

Sequences were edited (trimming and primer removal) on Geneious Prime version 2020.0.3 (www.geneious.com). Edited sequences were deposited in BOLD Systems (Ratnasingham and Hebert 2007), together with images, chromatograms and complementary information of the specimens (sampling site, date, taxonomy, store and institution information). Edited sequences were aligned using MAFFT 6.240 (Katoh et al. 2002) with the FFT-NS-i-x2 method and an unweighted pair group method with arithmetic mean (UPGMA) tree from distances corrected under a HKY distance model was generated. In addition, Maximum Likelihood (ML) phylogenetic analyses were run on the IQ-TREE web server at http://iqtree.cibiv.univie.ac.at (Trifinopoulos et al. 2016) using the best fitting substitution model for each codon partition as estimated with ModelFinder (Kalyaanamoorthy et al. 2017). Nodal support was obtained by 1,000 ultrafast boot-strap (UFBoot) replicates (Minh et al. 2013) (Suppl. material 2).

### Molecular species delimitation

Five different methods were applied for molecular species delimitation of the vouchers. First, the barcode index number (BIN) was implemented in the BOLD system. This approach provides an effective method for species delineation as each sequence cluster is assigned a unique alphanumeric (BIN URI, see Table 1), which reflects the patterning of intra- and interspecific divergences found in the overall BOLD database (Ratnasingham and Hebert 2007, but see Meier et al. (2022)). Second, the Assemble Species by Automatic Partitioning analysis (ASAP) was implemented. ASAP is a species delimitation method based on pairwise genetic distances ranked using a unique scoring system and consists of merging sequences into groups by an ascending hierarchical clustering until all sequences merge in a single group (Puillandre et al. 2020; https://bioinfo.mnhn.fr/abi/public/asap/). Analysis was implemented over the edited sequences using a K80 Kimura model and the lower ASAP scores as species delimiter parameter (Table 1). Third, we applied the Bayesian implementation of the Poisson tree processes model (PTP, Zhang et al. (2013); http://species.h-its.org/ptp/) by using the phylogenetic tree previously generated on IQ-tree. PTP works with a species delimitation hypothesis, based on the number of mutations (branch lengths) and evaluates species delimitation hypothesis using Maximum-Likelihood algorithms (mlPTP). Finally, as additional criteria for defining molecular entities, we also implemented 3% and 8% genetic distance thresholds using the distance-based UPGMA tree previously generated. The 3% threshold has been widely used to define OTUs in molecular studies (e.g. Hebert et al. (2003a), Magoga et al. (2021)), whereas the 8% threshold has been recently proposed as a conservative threshold in beetles (Salces-Castellano et al. 2021).

### Evaluating unrecorded and cryptic diversity within the Canary Islands context

To evaluate the previously unrecorded diversity in the Canary Islands that our dataset contains, we performed searches of the different species identified in our data within BIOTA. For each species within our dataset, we annotated the previously existing records and their status as endemic, native or potentially introduced taxa in the archipelago context.

To identify potential cryptic diversity within our dataset, we compare the morphological assignment provided to specimens with the results provided by the different species delimitation methods implemented. The number of morphological species that were split, merged or maintained was estimated for each delimitation method and the concordance amongst the specimens grouping resulting from each approach was evaluated.

### Evaluating unrecorded and cryptic diversity in the global context

To quantify the overall unrecorded mite diversity within our dataset, we used the Barcode of Life Data (BOLD) as the major source of barcode reference sequences available. BOLD contains 17,789,385 specimen records, of which 13,911,307 have barcode sequences (accessed at 02/08/2023). Of these, a total of 219,759 records are from mites, of which 181,682 have barcode sequences. Records with barcode sequence and identification to species level represent a total of 4,350 mite species, included in orders Holothyrida (1), Ixodida (334), Mesostigmata (992), Sarcoptiformes (769) and Trombidiformes (2,254). We have compared our sequences with those available on the platform to check the consistency of morphological identifications, detect potential cryptic diversity at the global scale and to investigate to what extent the diversity within our dataset is already represented in the BOLD repository.

First, we performed BOLD Identification System (IDS) (default setting parameters) for each sequence against the overall BOLD system (21/04/2023), including public and private barcode records. We extracted the taxonomic identification, based on morphology (species or genus level) and the similarity percentage to the best match using the BOLD Identification System (IDS) results for each sequence. Using this information: i) we estimated the overall similarity with BOLD sequences for each of our species and ii) we checked the coherence between the taxonomic identifications from BOLD and the morphological identifications of our specimens. In the cases where species-level identification agreed between both datasets, the overall similarity, the monophyly of the Canary Islands sequences and the geographical origin of BOLD sequences were evaluated to identify potential cryptic diversity within those mite species. Finally, as an additional indicator of how the diversity in our dataset is already reported within BOLD, the number of BINs that were only composed by our sequences (i.e. not including sequences already present on the repository) was recorded.

## Results

**Figure 1.**
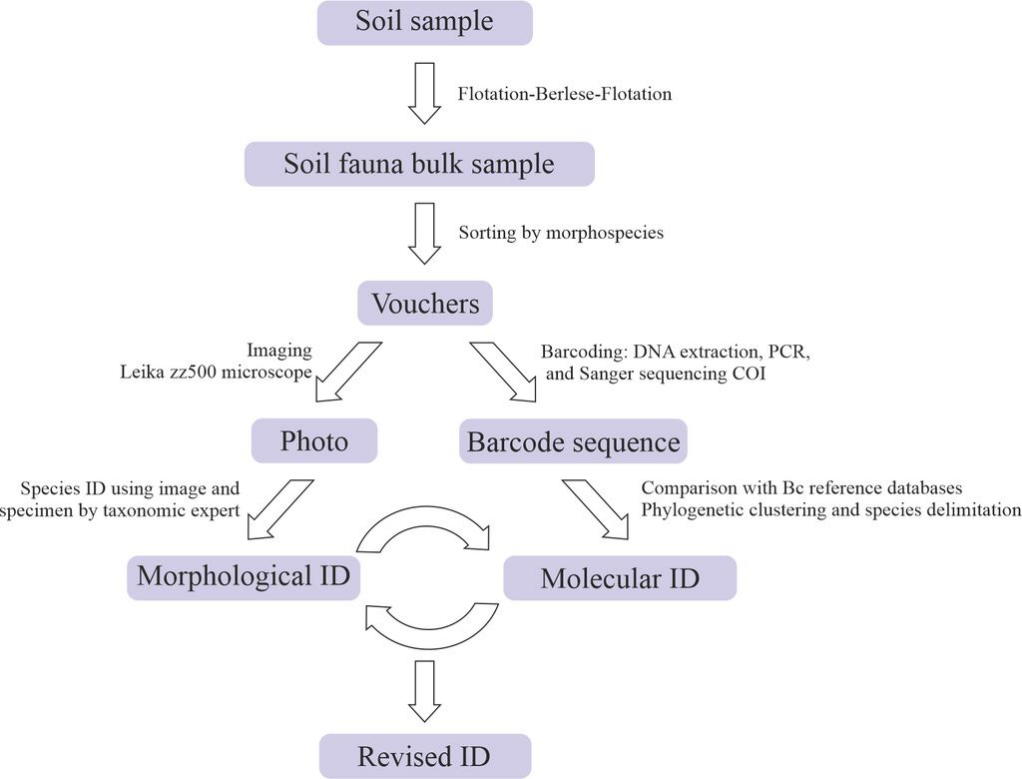
Schematic representation of the implemented workflow that combines traditional morphological identification and COI barcoding of soil arthropod specimens.

**Figure 2.**
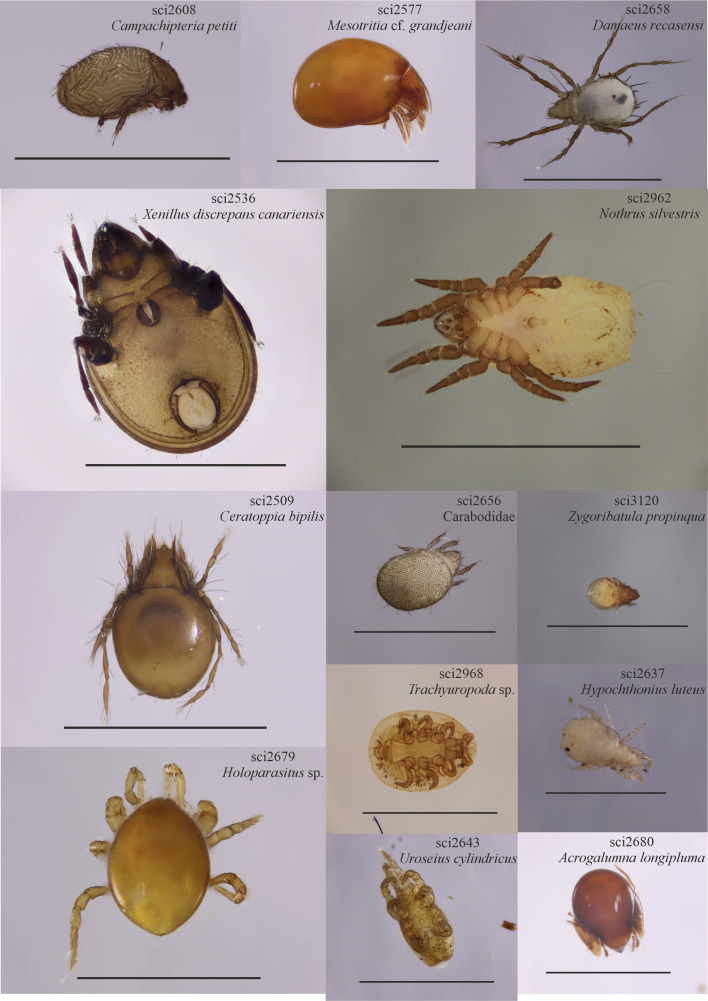
Representative examples of obtained images from voucher specimens. Scale = 1 mm. For specimen codes, see Suppl. materials 3, 4.

From the total 239 mite specimens selected, we recovered 168 barcode sequences, resulting in a success rate above 70%. Sequence lengths varied from 472 to 639 bp and included 153 haplotypes. The 168 barcoded specimens were morphologically identified as corresponding with 58 different morphological entities, of which 45 correspond to known species, four to unknown, likely new species of the genus *Xenillus* and nine entities (each represented by a single specimen) that could only be identified at the genus (six cases) or family (three cases) levels. Our barcoded dataset comprises entities from three orders (Mesostigmata, Sarcoptiformes and Trombidiformes) and 32 families, including Xenillidae (six entities), Phthiracaridae (five entities) and Euphthiracaridae and Oribatulidae (four entities each) as the four families with a higher number of species in our dataset (Table 1, Suppl. material 3). All sequences and associated metadata were included in the BOLD System within the project CISoilBiota, subproject CIACA (Acari of the Canary Islands) as part of the BOLD Campaign ‘Fauna of the Canary Islands’. Samples were named for the submission to BOLD Systems as CIACA001-21 to CIACA168-21 (see Suppl. material 4). See Figs 1, 2 for a schematic representation of the workflow and a subset of the photographic records uploaded to BOLD.

### Morphological and molecular species delimitations: evaluating cryptic diversity within the Canary Islands context

**Figure 3.**
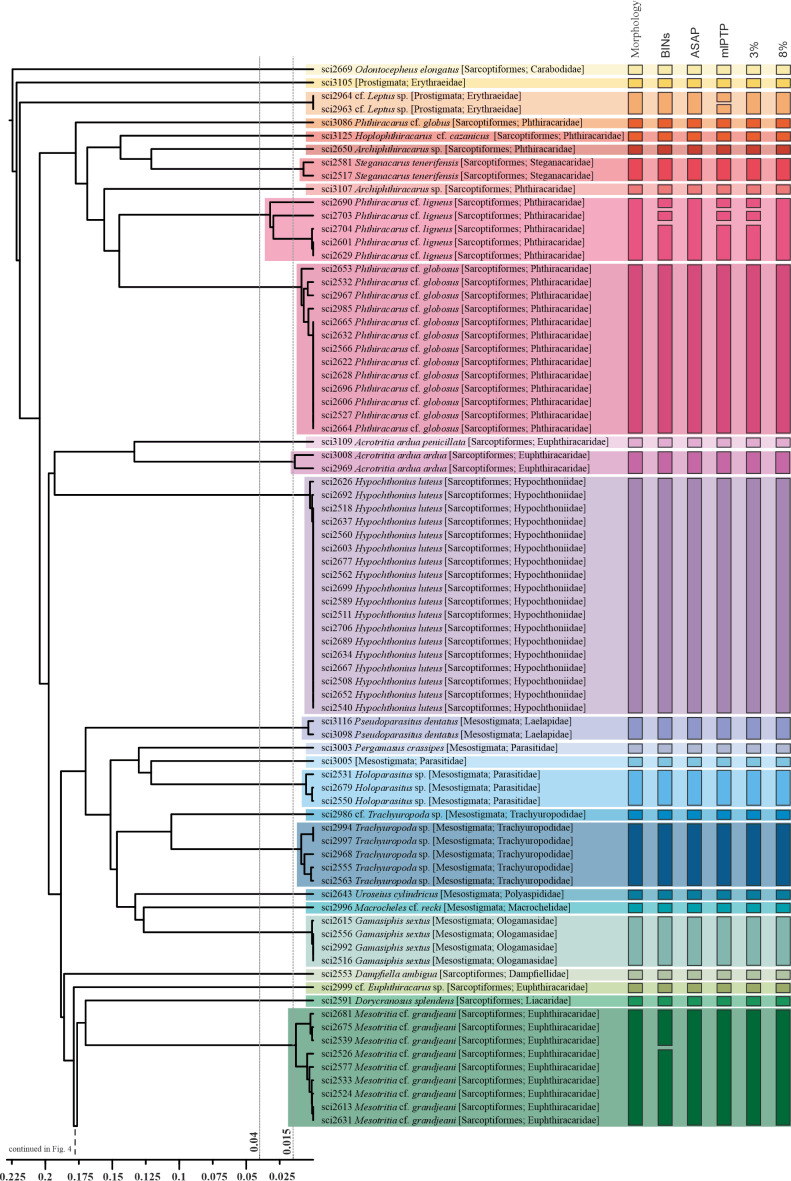
Distance-based UPGMA tree obtained using HKY corrected distances. Coloured horizontal blocks over the tree represent specimen clusters corresponding to morphological species. Vertical bars represent, from left to right: (i) morphological species, (ii) BINs classification in BOLD, (iii) species delimitation with ASAP, (iv) species delimitation with mlPTP, (v) 3% similarity clusters and (vi) 8% similarity clusters. At the bottom, each method's total number of species is presented. The X-axis represents genetic distance; with dotted lines corresponding 3% and 8% divergence thresholds (first half).

**Figure 4.**
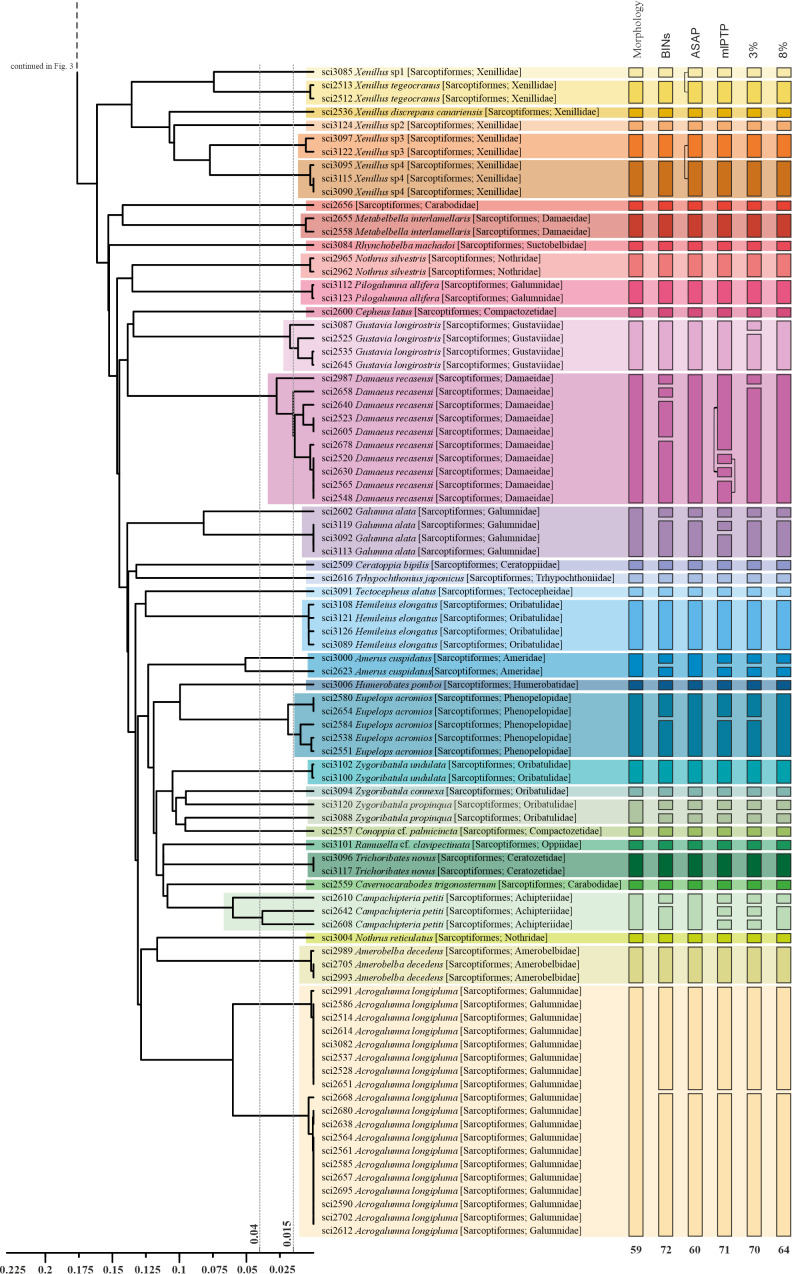
Distance-based UPGMA tree obtained using HKY corrected distances. Coloured horizontal blocks over the tree represent specimen clusters corresponding to morphological species. Vertical bars represent, from left to right: (i) morphological species, (ii) BINs classification in BOLD, (iii) species delimitation with ASAP, (iv) species delimitation with mlPTP, (v) 3% similarity clusters and (vi) 8% similarity clusters. At the bottom, each method's total number of species is presented. The X-axis represents genetic distance; with dotted lines corresponding 3% and 8% divergence thresholds (second half).

The different molecular species delimitation methods implemented resulted in a range from 60 to 85 molecular species delimited. The barcode index number (BIN) implemented in the BOLD System showed that our 168 voucher sequences were grouped in 71 BINs. Sixty-five of those BINs were newly generated by BOLD and assigned to sequences contributed in this study, while 11 sequences were grouped into six pre-existing BINs (access numbers in Table 1). ASAP analysis grouped our sequences in 60 molecular species according to the lower ASAP score, whereas PTP analysis delimited 71 molecular species for the mlPTP approach. Finally, the 3% genetic distance threshold resulted in 70 groups and the 8% genetic distance threshold in 64 (Figs 3, 4).

The concordance amongst the results of the different molecular delimitations implemented was relatively high. BINs, mPTP and 3% approaches resulted in a higher (but mostly concordant) number of delimited entities (Figs 3, 4). In all cases, molecular species delimitations exceeded the 58 entities morphologically delimited. In general, morphologically identified species and molecular species groupings were coincident, but at least eleven morphologically delimited species consistently included multiple molecular entities in at least one delimitation method (Phthiracaruscf.ligneus, Mesotritiacf.grandjeani, *Gustavialongirostris*, *Damaeusrecasensi*, *Galumnaalata*, *Ameruscuspidatus*, *Eupelopsacromios*, *Zygoribatulapropinqua*, *Campachipteriapetiti*, and *Acrogalumnalongipluma*) (Figs 3, 4).

### Comparison with the Biodiversity Databank of the Canary Islands: evaluating unrecorded diversity within the Canary Island context

**Figure 5.**
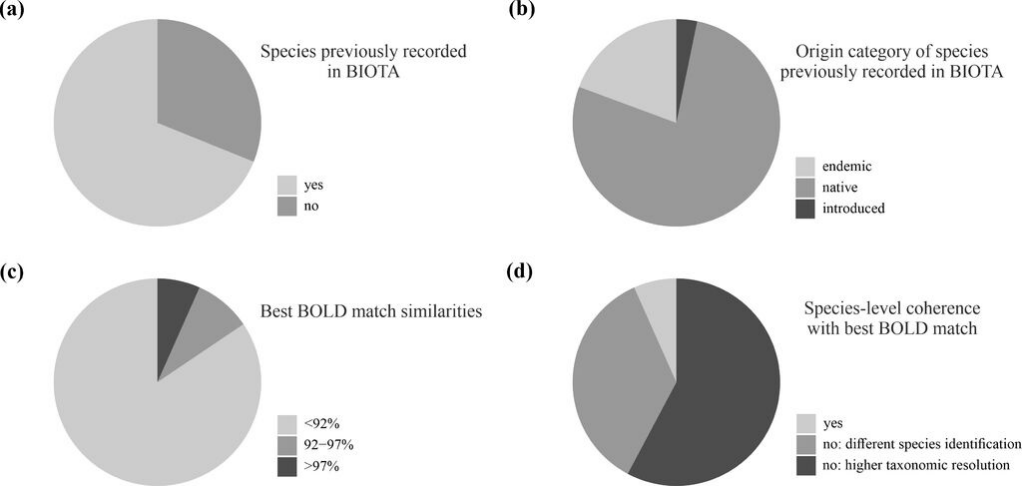
Evaluating unrecorded diversity within the Canary Islands and global context. **a** Proportion of species in our dataset that are already registered in the BIOTA database; **b** Category of origin (i.e. native non-endemic, introduced or endemic) for species recorded in BIOTA as reported within the database; **c** Similarity values of best matches of obtained sequences representing each species against BOLD Systems; **d** Species-level identification coherence between the specimens in our dataset and BOLD best matches. When there is no coherence, we specify if the BOLD best match was identified at species or higher taxonomic level (genus or family level).

Of the 45 morphological entities identified as known species in our dataset, 31 species (68%) were already registered as present in the archipelago, whereas the remaining 14 (31%) represented new species and genera records at the archipelago level (Table 1, Fig. 5a).

Of the 31 species in our dataset that are present in the BIOTA, six species are endemic to the Islands (19%) and the rest are considered non-endemic native species (24 species, 77%) or introduced species (one species, 3%) (Table 1, Fig. 5b).

### Comparison with the BOLD system: Evaluating unrecorded and cryptic diversity in the global context

BOLD Identification System (IDS) results for the 168 sequences in our dataset resulted in a range of similarity percentages, with the best matches ranging from 74.48% to 99.5%. Amongst the 45 morphological entities identified as known species, 38 (84%) reported a similarity below 92%, four (9%) from 92% to 97% and three (7%) above 97% (Table 1, Fig. 5c). Regarding the taxonomic identity of the BOLD best matches for our sequences, the best match has no species-level identification for 26 (58%) entities. For the remaining cases where best BOLD matches have species-level identifications, three (6%) are concordant with the morphological identifications of our specimens and 16 (35%) showed no concordant species-level identifications (Table 1, Fig. 5d).

The only cases where species-level identification agrees with the BOLD dataset are *Acrogalumnalongipluma*, *Hypochthoniusluteus* and *Odontocepheuselongatus*. In the case of *Acrogalumnalongipluma*, the overall similarity of Canarian specimens with BOLD sequences was 96.45%. In the case of *Hypochthoniusluteus*, the overall similarity of our Canarian specimens with the only one registered on BOLD was 96.34%. Finally, in the case of *Odontocepheuselongatus*, the overall similarity of Canarian specimens with BOLD sequences was 74.48%.

## Discussion

### A molecular barcoding framework for the soil fauna of the Canary Islands

Our study initiates the Barcode Database of soil fauna from the Canary Islands (CISoilBiota) by developing a standardised workflow that combines specific soil sampling, Berlese extraction, sample sorting, COI barcoding and traditional taxonomic identification of barcoded specimens. The workflow has been applied to 239 mite specimens, of which we recovered 168 sequences. This represents a success of 70%, similar to success rates in other barcoding studies (e.g. deWaard (2019), Salces-Castellano et al. (2021), Suárez et al. (2022), Suárez et al. (2023), Caterino and Recuero (2023)). We did not detect any pattern amongst failures regarding taxonomical assignments or geographic distribution of soil samples. Causes of failures may be indicative of a low quantity of DNA retrieved, considering that we worked with minute mesofauna specimens or poor quality of DNA, as specimens were collected using Berlese apparatus with water on the collecting recipients. Each Berlese apparatus was revised and specimens were transferred to ethanol every two days to minimise the degradation of DNA. Still, we cannot discard DNA degradation as affecting PCR performance in some cases. Although extracted DNA quantification and further dilutions or reconcentration will help to obtain a higher success rate, we consider this 70% success rate as a good starting point for further development, considering inherent difficulties of DNA work with small-sized soil mesofauna.

One of these difficulties is the incompatibility of the procedures used for the morphological study of these minute organisms (requiring microscopic preparations where specimens are cleared and fixed with different chemical products) and DNA preservation. Here, we solve that by implementing a protocol of specimen imaging and non-destructive DNA extractions for mites that allow the morphological study of the specimens after DNA extraction. Our results demonstrated that non-destructive DNA extraction of soil mites is feasible without compromising the morphological integrity of specimens.

Another difficulty in implementing barcoding to soil mesofauna is associated with the reduced body size of specimens and the low DNA concentration retrieved. The DNA extraction and PCR protocol performed here appears adequate under these low DNA conditions. DNA extraction was implemented using a magnetic-bead approach in a robotic platform; this semi-automated approach is optimal for implementing arthropod barcoding because it facilitates the standardised processing of high numbers of specimens while maximising the quality of DNA extracts for long-term storage (Arribas et al. 2022). We reduced reagent volumes in half without an evident impact on DNA extraction performance. Further tests with more reduced volumes would be desirable to minimise costs associated with DNA extraction. Finally, the high phylogenetic diversity within soil mites could challenge the selection of the primer sets for PCR amplification of the barcode fragment. Our results, aligned with previous studies (Young et al. 2012, Arribas et al. 2016, Arribas et al. 2019), demonstrate an overall good performance of the Folmer degenerate barcode primers (Yu et al. 2012) for the broad diversity of soil mites.

We expect that barcoding effort over soil mites can be additionally improved by the application of High Throughput Sequencing (HTS) approaches, at the same time that costs are reduced (Srivathsan et al. 2018, Creedy et al. 2021, Srivathsan et al. 2021, Emerson et al. 2022). Although Sanger sequencing is an efficient and optimised technique, multiplexing approaches combined with the strength of HTS can be used to generate thousands of barcode sequences in a faster and cheaper way (Srivathsan et al. 2019, Srivathsan et al. 2023, Vasilita et al. 2023). The rigorous implementation of HTS barcoding methodologies on the remarkably abundant and hyperdiverse soil mesofauna of arthropods holds great promise in addressing the global lack of knowledge on soil biodiversity. Still, to maximise the utility of obtained sequences as barcode references, protocols as proposed here, are fundamental, as the link amongst obtained sequences, properly preserved voucher specimens and images is maintained. These procedures include costly and time-consuming steps for specimen sorting, imaging and puncturing before DNA extraction, in addition to requiring great taxonomic expertise. We acknowledge that morphological identification is the main bottle-neck for the whole approach and, in agreement with Srivathsan et al. (2021), we envision a system where HTS barcoding can be used to obtain barcode sequences from high numbers of soil mesofauna specimens that, after the application of DNA similarity clustering and molecular identification methods, can be subsampled for a detailed morphological study of a much-reduced number of representative specimens.

### Barcoding to unveil cryptic diversity in soil fauna

The molecular species delimitations showed broad consistency amongst them and with morphological species identifications in our dataset (Figs 3, 4). ASAP and 8% threshold showed a higher agreement in delimited entities between them and with the morphological identification, whereas BINs, mlPTP and 3% resulted in additional splits for some lineages, likely representing over-splitting as previously reported in other studies (Copilaş-Ciocianu et al. 2022, Ranasinghe et al. 2022). Still, the comparison of the more conservative ASAP and 8% threshold with the morphological identification allowed us to detect several cases of inconsistency and potential cryptic diversity. The taxonomic challenge posed by cryptic species (two or more morphologically similar species classified as a single species) has been recognised as a significant limitation in quantifying soil mesofauna (Emerson et al. 2011, Pfingstl et al. 2021, Szudarek-Trepto et al. 2021, Yin et al. 2022). Different studies, implementing DNA sequencing, have revealed that cryptic diversity could be massive in specific soil lineages (Cicconardi et al. 2010, Cicconardi et al. 2013). In this study, despite our reduced sampling (168 barcodes from mite specimens), we evidenced several examples of potential cryptic diversity within the mites of the Canary Islands, where molecular delimitations identify multiple divergent lineages within a single morphological species.

We have detected a series of cases where specimens, morphologically identified as a species, show intraspecific divergences higher than 3%. Part of these cases consists of monophyletic lineages where internal divergences are higher than 3%, but lower than 8%. Here we found the cases of: (i) Phthiracaruscf.ligneus with three lineages with divergences over 6%; (ii) *Damaeusrecasensi* with two lineages (one with a single specimen) with divergences above 5%; (iii) *Eupelopsacromios* with two lineages with divergences above 3%; and (iv) *Gustavialongirostris* with two lineages with divergences above 3%. In these four cases, the moderately high intraspecific divergences found are compatible with a single species, which is also suggested by molecular species delimitation methods, such as ASAP (Figs 3, 4). In fact, high intraspecific variation can be expected for soil mites if we consider the huge population sizes reported for some soil taxa (Petersen and Luxton 1982, Endlweber et al. 2006) and the expectations from the neutral theory of molecular evolution, with genetic diversity increasing with a larger effective population size and the decreasing effects of drift (Kimura 1979). Still, these divergences also suggest that additional attention should be placed on these lineages, as they likely represent native species within the Canaries, which is a highly fragmented landscape that can contribute to geographic isolation and diversification (Juan et al. 2000). Alternatively, this pattern may also reflect the human-mediated introduction of new populations for Canarian native species or even cases of multiple introduction of non-native species. More detailed studies will be required to distinguish amongst the different alternatives for each lineage.

We have also detected other cases where specimens classified as a single species are split into different lineages with divergences higher than 8% for the barcode fragment (Figs 3, 4). These cases include: (i) *Campachipteriapetiti*, represented by three specimens with divergences between 7.1 and 11.1%; (ii) *Galumnaalata* with two lineages diverging 14.3%; (iii) *Ameruscuspidatus* represented by only two specimens having a divergence of 9.3%; (iv) *Acrogalumnalongipluma* with two lineages with divergences of 11.5%; (v) *Zygoribatulapropinqua* represented by only two specimens having a divergence of 17.5%; (vi) *Acrotritiaardua*, where the two morphological subspecies show molecular divergences of 21.7% and (vii) the two morphologically highly similar specimens classified as *Archiphthiracarus* sp., which are only distantly related phylogenetically (26.9% divergence). All these cases suggest speciation with reduced morphological differentiation, although deep mitochondrial DNA divergence has been also shown to not indicate distinct species in some lineages (e.g. Leo et al. (2010)). A detailed morphological study and the sequencing of additional genes from the nuclear genome and specimens from a wider geographical range will be needed to clarify their taxonomic status.

Further implementation of the proposed barcoding workflow within the Canary Islands will contribute to elucidating the status of the reported cases and, presumably, to detect additional cases of cryptic diversity. An integrative approach, with parallel and interactive morphological and molecular work, will contribute to accelerating species inventory and discovery. For example, in our dataset and within the genus *Xenillus*, two already-described species are detected, with additional specimens showing morphological variation not matching any described species. Molecular analysis shows consistent results with morphology, suggesting the existence of four additional new species within the genus with divergences above 13.2% (Figs 3, 4).

Beyond the prevalence of cryptic diversity within the soil mites of the Canary Islands, our results point to the generality of this pattern globally. The analyses comparing our sequences with the BOLD database found three paradigmatic cases of potential cryptic diversity within worldwide distributed species. The first one is the case of *Acrogalumnalongipluma*, with available barcode sequences from Canada, Germany, Finland, UK and the Canary Islands, forming five differentiated geographically coherent lineages with similarities below 97%. Of these, two lineages are exclusively found in the Canary Islands with divergences higher than 12% and not showing a sister taxa relationship (Suppl. materials 5, 6). This pattern suggests that, under the taxonomic name *Acrogalumnalongipluma*, we currently enclose a complex of species, that could include two endemic species for the Canary Islands and that requires a detailed morphological revision. The second case is that of *Hypochthoniusluteus*, where the Canarian specimens form a lineage sister to a specimen from Belgium, with an overall similarity of 96.34%, again pointing to potential cryptic diversity within this morphologically described species (Suppl. material 7). In the third case, that of *Odontocepheuselongatus*, available sequences from Norway, Finland and the Canary Islands form three differentiated lineages with similarities below 75%, one of these lineages exclusively composed of Canarian specimens, showing again cryptic diversity within a highly similar morphology (Suppl. material 8).

### Barcoding to unveil the (unknown) diversity of soil fauna within the Canary Islands

If a reference database is poorly populated for a specific group, the probability of inacurate taxonomic assignment is higher and placement to high taxonomic ranks is frequent (Somervuo et al. 2017). Our results illustrate the high magnitude of unrecorded diversity within soil mites and associated issues when comparing obtained barcodes with reference databases. Most of the taxa within our dataset were not previously represented in the BOLD database, with most generated barcode sequences showing low similarity values with the best available matches in BOLD. In addition, a high proportion of best matches corresponded to specimens without a species-level identification in BOLD (26 cases) or to different species to those here identified morphologically (16 cases, all with low similarity). The lack of morphological identifications to the species level can be understood given: (i) the great species richness, abundance and local-scale heterogeneity of mesofauna communities, (ii) the poor taxonomic background knowledge of many soil lineages and geographic areas and (iii) the general lack of taxonomic expertise on soil mites (André et al. 1994, Decaëns 2010, Cameron et al. 2018, Guerra et al. 2020, White et al. 2020). Consequently, species widely distributed, naturally or by mean of human introductions, are likely better represented in public repositories. In contrast, those species locally endemic with small distributional ranges are represented to a lesser extent and only from a few geographical regions (Porter and Hajibabaei 2018). This lack of representation and taxonomic resolution in reference databases limits their potential to provide a reliable taxonomic assignment to newly-generated barcodes, highlighting the imperative need for further barcode projects on soil fauna integrating taxonomic expertise.

Within the context of the Canary Islands, our results point to a massive under-representation of the diversity of soil mites in biodiversity databases. The BIOTA database contains records for 474 species and subspecies of mites, of which 425 are considered native species and 49 introduced species. Of those species classified as native, 110 species are considered endemic to the Canary Islands and 104 species endemic to the Macaronesian Region. Regarding our data, 14 of the 45 (31%) species, for which we obtained a species-level identification, represent the first record for the Canary Islands, all of them also providing the first record at the genus-level. All of these are species known from outside the Canaries and are now reported to the Canaries for the first time. These species may correspond to native non-endemic species or introduced species, according to their known distribution outside the Canaries, but given the absence of reference sequences for most of these species, we cannot discard that they represent additional cases of cryptic diversity. For example, in our dataset, we found four cases in which, although it is not the best match in BOLD, there has been a match with a sequence of the same species. *Acrotritiaarduaardua* and *A.penicillata* have a 78.86-79.57% similarity with several sequences named *Rhysotritiaardua* (junior synonym) from Canada, Poland and Norway (see Suppl. materials 9, 10). Phthiracaruscf.globosus has a 77.78-77.89% similarity with sequences from Finland (see Suppl. material 11). *Xenillustegeocranus* has a 78.15-80.07% similarity with sequences from Canada, Finland, Norway and Slovakia (see Suppl. material 12). These worldwide matches suggest that species that have been taxonomically assigned to a single species may constitute cryptic lineages. To advance our knowledge of the magnitude of biodiversity and level of endemicity of the Canary Islands, we will need further efforts in generating barcode reference sequences, with reliable taxonomic identification, from inside the Canaries, but also from other regions. Barcoding specimens unambiguously associated with a particular species considering both its morphology and geographic origin is needed. These data will be key to distinguishing amongst native, endemic and introduced species within the Canaries, allowing us to generate reliable local inventories of soil fauna.

## Conclusions

This study provides and demonstrates the efficiency of a standardised workflow that combines traditional morphological identification and COI barcoding for the challenging soil fauna of mites within the framework of the BOLD System. Despite our reduced sampling, our results on interrogating the generated biodiversity data demonstrate the remarkable unrecorded mesofauna diversity present in the soils of the archipelago. This study represents the first attempt to document COI barcodes for soil mesofauna in the Canary Islands and provides the basis for the Canary Islands Soil Biodiversity barcode database (CISoilBiota). The wider implementation of this barcoding workflow within the Canaries holds the promise for a massive biodiversity discovery.

## Supplementary Material

2FCFA752-1AD6-5B03-8A5D-A9B176E5546F 10.3897/BDJ.12.e113301.suppl1 Supplementary material 1 Table S1 Data type Dataset (excel table) Brief description Sampling localities, with data on habitat type, coordinates, altitude and date of collection. File: oo_908848.xlsx https://binary.pensoft.net/file/908848 Irene Santos-Perdomo, Daniel Suárez, María L. Moraza, Paula Arribas, Carmelo Andújar

0C2632DD-2DED-5267-B8BD-40226EA99620 10.3897/BDJ.12.e113301.suppl2 Supplementary material 2 Figure S1 Data type Image (TIFF) Brief description Maximum Likelihood (ML) phylogenetic tree obtained for the barcode dataset with IQ-TREE using the best fitting substitution model for each codon partition and nodal support obtained by 1,000 ultrafast boot-strap replicates. File: oo_908800.tif https://binary.pensoft.net/file/908800 Irene Santos-Perdomo, Daniel Suárez, María L. Moraza, Paula Arribas, Carmelo Andújar

461DE84A-19F2-53A0-9668-B4B82B86FC1C 10.3897/BDJ.12.e113301.suppl3 Supplementary material 3 Table S2 Data type Dataset (excel table) Brief description Specimens morphological identification at order, family, genus and species levels. File: oo_956844.xlsx https://binary.pensoft.net/file/956844 Irene Santos-Perdomo, Daniel Suárez, María L. Moraza, Paula Arribas, Carmelo Andúja

67163793-EB6C-5735-89F0-251B19E6BA1A 10.3897/BDJ.12.e113301.suppl4 Supplementary material 4 Table S3 Data type Dataset (excel table) Brief description Data of studied specimens within the BOLD project CISoilBiota, subproject CIACA (Acari of the Canary Islands). File: oo_908851.xlsx https://binary.pensoft.net/file/908851 Irene Santos-Perdomo, Daniel Suárez, María L. Moraza, Paula Arribas, Carmelo Andújar

C56543DE-FE3A-55C0-AD32-43D73DF2422C 10.3897/BDJ.12.e113301.suppl5 Supplementary material 5 Figure S2a Data type Phylogenetic tree in PDF format Brief description Distance-based phylogenetic tree generated by BOLD using K2P corrected distances including best BOLD matches of *Acrogalumnalongipluma* (lineage A). File: oo_908853.pdf https://binary.pensoft.net/file/908853 Irene Santos-Perdomo, Daniel Suárez, María L. Moraza, Paula Arribas, Carmelo Andújar

6FF536C3-783B-5192-B7AC-350ED3A863EE 10.3897/BDJ.12.e113301.suppl6 Supplementary material 6 Figure S2b Data type Phylogenetic tree in PDF forma Brief description Distance-based phylogenetic tree generated by BOLD using K2P corrected distances including best BOLD matches of *Acrogalumnalongipluma* (lineage B). File: oo_908857.pdf https://binary.pensoft.net/file/908857 Irene Santos-Perdomo, Daniel Suárez, María L. Moraza, Paula Arribas, Carmelo Andújar

996FA055-DFA1-5094-B530-F5B8222B0814 10.3897/BDJ.12.e113301.suppl7 Supplementary material 7 Figure S3 Data type Phylogenetic tree in PDF format Brief description Distance-based phylogenetic tree generated by BOLD using K2P corrected distances including best BOLD matches of *Hypochthoniusluteus*. File: oo_908858.pdf https://binary.pensoft.net/file/908858 Irene Santos-Perdomo, Daniel Suárez, María L. Moraza, Paula Arribas, Carmelo Andújar

CC075F80-EECB-5FA1-B006-39F295FEF6BF 10.3897/BDJ.12.e113301.suppl8 Supplementary material 8 Figure S4 Data type Phylogenetic tree in PDF format Brief description Distance-based phylogenetic tree generated by BOLD using K2P corrected distances including best BOLD matches of *Odontocepheuselongatus*. File: oo_908859.pdf https://binary.pensoft.net/file/908859 Irene Santos-Perdomo, Daniel Suárez, María L. Moraza, Paula Arribas, Carmelo Andújar

BE5F5E8C-FE5D-553A-8165-66435EA36BD8 10.3897/BDJ.12.e113301.suppl9 Supplementary material 9 Figure S5a Data type Phylogenetic tree in PDF format Brief description Distance-based phylogenetic tree generated by BOLD using K2P corrected distances including best BOLD matches of *Acrotritiaarduaardua*. File: oo_908861.pdf https://binary.pensoft.net/file/908861 Irene Santos-Perdomo, Daniel Suárez, María L. Moraza, Paula Arribas, Carmelo Andújar

E136182D-6BD9-5F8F-A744-92829E65229F 10.3897/BDJ.12.e113301.suppl10 Supplementary material 10 Figure S5b Data type Phylogenetic tree in PDF format Brief description Distance-based phylogenetic tree generated by BOLD using K2P corrected distances including best BOLD matches of *Acrotritiapenicillata*. File: oo_908865.pdf https://binary.pensoft.net/file/908865 Irene Santos-Perdomo, Daniel Suárez, María L. Moraza, Paula Arribas, Carmelo Andújar

1FA8BC53-4F47-51DF-80A2-2C5586BF1360 10.3897/BDJ.12.e113301.suppl11 Supplementary material 11 Figure S6 Data type Phylogenetic tree in PDF format Brief description Distance-based phylogenetic tree generated by BOLD using K2P corrected distances including best BOLD matches of *Phthiracaruscfglobosus*. File: oo_908866.pdf https://binary.pensoft.net/file/908866 Irene Santos-Perdomo, Daniel Suárez, María L. Moraza, Paula Arribas, Carmelo Andújar

D35B214C-2100-5FF2-A9BE-0104226A976C 10.3897/BDJ.12.e113301.suppl12 Supplementary material 12 Figure S7 Data type Phylogenetic tree in PDF format Brief description Distance-based phylogenetic tree generated by BOLD using K2P corrected distances including best BOLD matches of *Xenillustegeocranus*. File: oo_908867.pdf https://binary.pensoft.net/file/908867 Irene Santos-Perdomo, Daniel Suárez, María L. Moraza, Paula Arribas, Carmelo Andújar

## Figures and Tables

**Table 1.** Species-level inventory in our dataset, including: morphological identification, number of barcoded sequences, entities delimited for each species delimitation methods (new generated BINs are highlighted by an ‘*’), representation in BIOTA database (and origin category), similarity percentage with best BOLD match and coherence between species-level identification and best BOLD match identification.

**MORPHOLOGICAL IDENTIFICATION**	**Nº OF SEQUENCES**	**BIN**	**ASAP**	**mlPTP**	**BIOTA MATCH**	**NEAREST BOLD MATCH**	**BOLD COHERENCE**
**Order Mesostigmata**							
Fam. Laelapidae							
*Pseudoparasitusdentatus* (Halbert, 1920)	2	BOLD:AEI7729*	ASAP21	mlPTP8	yes/native	*Ornithonyssussylviarum* (79.44%)	no: different species identification
Fam. Macrochelidae							
Macrocheles (Macrholaspis) cf. recki Bregetova & Koroleva, 1960	1	BOLD:AEI5961*	ASAP54	mlPTP17	yes/native	Mesostigmata (81.9%)	no: higher taxonomic resolution
Fam. Ologamasidae							
*Gamasiphissextus* Vitzthum, 1921	4	BOLD:AEI1055*	ASAP13	mlPTP18	no	*Gamasiphis* sp. JCS03 (83.72%)	no: higher taxonomic resolution
Fam. Parasitidae							
*Holoparasitus* sp.	3	BOLD:AEI8993*	ASAP6	mlPTP23	NA	Parasitidae (83.00%)	NA
Parasitidae	1	BOLD:AEI6503*	ASAP39	mlPTP22	NA	*Poecilochirus* (84.57%)	NA
*Pergamasuscrassipes* (Linnaeus, 1758)	1	BOLD:ACQ8500	ASAP2	mlPTP21	yes/introduced	Mesostigmata (98.34%)	no: higher taxonomic resolution
Fam. Polyaspididae							
*Uroseiuscylindricus* (Berlese, 1916)	1	BOLD:AEI1534*	ASAP44	mlPTP45	no	*Polyaspinushigginsi* (84.23%)	no: different species identification
Fam. Trachyuropodidae							
cf. *Trachyuropoda* sp.	1	BOLD:ADH9050	ASAP35	mlPTP43	NA	Uropodidae (99.5%)	NA
*Trachyuropoda* sp.	5	BOLD:AAZ2213	ASAP1	mlPTP44	NA	Mesostigmata (98.54%)	NA
**Order Trombidiformes**							
Fam. Erythraeidae							
cf. *Leptus* sp.	2	BOLD:AEI0533*	ASAP47	mlPTP66; mlPTP67	NA	Arachnida (88.08%)	NA
Erythraeidae	1	BOLD:AEI6506*	ASAP38	mlPTP7	NA	Trombiculidae (78.51%)	NA
**Order Sarcoptiformes**							
Fam. Achipteriidae							
*Campachipteriapetiti* (Travé, 1960)	3	BOLD:AEI0246*; BOLD:AEI6330*	ASAP20	mlPTP54; mlPTP59; mlPTP60	no	Achipteriidae (86.7%)	no: higher taxonomic resolution
Fam. Ameridae							
*Ameruscuspidatus* (Berlese, 1883)	2	BOLD:AEI0247*; BOLD:AEI4260*	ASAP23	mlPTP57; mlPTP58	yes/native	*Ceratozetesgracilis* (83.02%)	no: different species identification
Fam. Amerobelbidae							
*Amerobelbadecedens* Berlese, 1908	3	BOLD:AEI8991*	ASAP7	mlPTP38	yes/native	*Eueremaeus* (83.33%)	no: higher taxonomic resolution
Fam. Carabodidae							
Carabodidae	1	BOLD:AEI9656*	ASAP40	mlPTP29	NA	Hermanniellidae (80.95%)	NA
*Cavernocarabodestrigonosternum* (Pérez-Íñigo, 1976)	1	BOLD:AEI0245*	ASAP53	mlPTP37	yes/endemic	*Oribatodesmirabilis* (85.96%)	no: different species identification
*Odontocepheuselongatus* (Michael, 1879)	1	BOLD:AEI0250*	ASAP60	mlPTP2	yes/native	*Odontocepheuselongatus* (74.48%)	yes
Fam. Ceratoppiidae							
*Ceratoppiabipilis* (Hermann, 1804)	1	BOLD:AEH9721*	ASAP49	mlPTP34	yes/native	*Ceratoppia* (82.96%)	no: higher taxonomic resolution
Fam. Ceratozetidae							
*Trichoribatesnovus* (Sellnick, 1928)	2	BOLD:AEI6507*	ASAP41	mlPTP46	no	*Oribatella* (84.86%)	no: higher taxonomic resolution
Fam. Compactozetidae							
*Cepheuslatus* Koch, 1835	1	BOLD:AEI5729*	ASAP55	mlPTP36	yes/native	Neoliodidae (82.41%)	no: higher taxonomic resolution
Conoppiacf.palmicincta (Michael, 1884)	1	BOLD:AEI8929*	ASAP46	mlPTP48	yes/native	*Eremaeus* (88.68%)	no: higher taxonomic resolution
Fam. Damaeidae							
*Damaeusrecasensi* Capilla, 1971	10	BOLD:AEI4384*; BOLD:AEI4385*; BOLD:AEI4386*; BOLD:AEI7071*	ASAP14	mlPTP70; mlPTP71	yes/native	*Epidamaeus* (83.67%)	no: higher taxonomic resolution
*Metabelbellainterlamellaris* Pérez-Íñigo, 1987	2	BOLD:AEI0249*	ASAP45	mlPTP24	yes/native	Damaeidae (80.81%)	no: higher taxonomic resolution
Fam. Dampfiellidae							
*Dampfiellaambigua* Pérez-Íñigo, 1976	1	BOLD:AEI5421*	ASAP50	mlPTP1	yes/endemic	*Baryscapusservadeii* (77.57%)	no: different species identification
Fam. Euphthiracaridae							
*Acrotritiaarduaardua* (Koch, 1841)	2	BOLD:AAF9157	ASAP57	mlPTP33	yes/native	Euphthiracaridae (95.34%)	no: higher taxonomic resolution
*Acrotritiapenicillata* (Pérez-Íñigo, 1969)	1	BOLD:ADX1060	ASAP30	mlPTP32	no	Sarcoptiformes (99.5%)	no: higher taxonomic resolution
cf. *Euphthiracarus* sp.	1	BOLD:AEH9008*	ASAP56	mlPTP14	NA	*Euphthiracarusmonodactylus* (81.03%)	NA
Mesotritiacf.grandjeani (Feider & Suciu, 1957)	9	BOLD:AEI8467*; BOLD:AEI6713*	ASAP22	mlPTP3	no	Arthropoda (78.86%)	no: higher taxonomic resolution
Fam. Galumnidae							
*Acrogalumnalongipluma* (Berlese, 1904)	19	BOLD:AEI1056*; BOLD:AEI5290*	ASAP9; ASAP10	mlPTP55; mlPTP56	yes/native	*Acrogalumnalongipluma* (96.45%)	yes
*Galumnaalata* (Hermann, 1804)	4	BOLD:AEI4158*; BOLD:AEI8205*	ASAP17; ASAP18	mlPTP49; mlPTP68; mlPTP69	yes/native	*Eupelops* (82.09%)	no: higher taxonomic resolution
*Pilogalumnaallifera* (Oudemans, 1919)	2	BOLD:AEH9722*	ASAP31	mlPTP19	yes/endemic	*Cepheus* (82.72%)	no: higher taxonomic resolution
Fam. Gustaviidae							
*Gustavialongirostris* Mihelcic, 1957	4	BOLD:AEI3725*	ASAP33	mlPTP26	no	*Chamobatescuspidatus* (81.92%)	no: different species identification
Fam. Humerobatidae							
*Humerobatespomboi* Pérez-Íñigo, 1992	1	BOLD:AEI4261*	ASAP29	mlPTP47	yes/native	Humerobatidae (86.53%)	no: higher taxonomic resolution
Fam. Hypochthoniidae							
*Hypochthoniusluteus* Oudemans, 1917	18	BOLD:AEI3587*	ASAP5	mlPTP13	yes/native	*Hypochthoniusluteus* (96.34%)	yes
Fam. Liacaridae							
*Dorycranosussplendens* (Coggi, 1898)	1	BOLD:AEI6712*	ASAP26	mlPTP4	yes/native	Oppiidae (76.08%)	no: higher taxonomic resolution
Fam. Nothridae							
*Nothrusreticulatus* Sitnikova, 1975	1	BOLD:AEI6500*	ASAP43	mlPTP39	no	*Nothrus* (95.27%)	no: higher taxonomic resolution
*Nothrussilvestris* Nicolet, 1855	2	BOLD:AEI0848*	ASAP28	mlPTP40	yes/native	*Nothrus* (82.57%)	no: higher taxonomic resolution
Fam. Oppiidae							
Ramusellacf.clavipectinata (Michael, 1885)	1	BOLD:AEI7727*	ASAP32	mlPTP28	yes/native	*Eremaeus* (84.67%)	no: higher taxonomic resolution
Fam. Oribatulidae							
*Hemileiuselongatus* E.Pérez-Íñigo, 1978	4	BOLD:AEI4159*	ASAP8	mlPTP20	yes/native	*Hemileiusinitialis* (85.82%)	no: different species identification
*Zygoribatulaconnexa* (Berlese, 1904)	1	BOLD:AEI7730*	ASAP16	mlPTP25	yes/native	*Oribatulatibialis* (85.02%)	no: different species identification
*Zygoribatulapropinqua* (Oudemans, 1902)	2	BOLD:AEI7725*; BOLD:AEI8260*	ASAP34; ASAP59	mlPTP27	yes/native	*Eueremaeussilvestris* (84.85%)	no: different species identification
*Zygoribatulaundulata* Berlese, 1916	2	BOLD:AEI7728*	ASAP12	mlPTP31	yes/native	*Achipteriacoleoptrata* (86.16%)	no: different species identification
Fam. Phenopelopidae							
*Eupelopsacromios* (Hermann, 1804)	5	BOLD:AEI2704*; BOLD:AEI3535*	ASAP36	mlPTP64; mlPTP65	yes/native	*Eupelops* (84.57%)	no: higher taxonomic resolution
Fam. Phthiracaridae							
*Archiphthiracarus* sp.	2	BOLD:AEI0248*; BOLD:AEI7726*	ASAP42; ASAP58	mlPTP10; mlPTP16	NA	*Phthiracarusglobosus* (95.78%)	NA
Hoplophthiracaruscf.cazanicus Feider & Calugar, 1970	1	BOLD:AEH8933*	ASAP24	mlPTP15	no	*Austrophthiracaruscostai* (80.35%)	no: different species identification
Phthiracaruscf.globosus (Koch, 1841)	13	BOLD:AEI2040*	ASAP11	mlPTP11	no	*Phthiracarus* (79.94%)	no: higher taxonomic resolution
Phthiracaruscf.globus Parry, 1979	1	BOLD:AEI5730	ASAP25	mlPTP9	no	*Phthiracarus* (98.04%)	no: higher taxonomic resolution
Phthiracaruscf.ligneus Willmann, 1931	5	BOLD:AEH9005*; BOLD:AEI6501*; BOLD:AEI7167*	ASAP27	mlPTP61; mlPTP62; mlPTP63	no	*Phthiracarusglobosus* (78.43%)	no: different species identification
Fam. Steganacaridae							
*Steganacarustenerifensis* Pérez-Íñigo, 1972	2	BOLD:AEI8994*	ASAP19	mlPTP12	yes/endemic	*Steganacarusmagnus* (84.21%)	no: different species identification
Fam. Suctobelbidae							
*Rhynchobelbamachadoi* Pérez-Íñigo, 1976	1	BOLD:AEI6502*	ASAP48	mlPTP6	yes/endemic	*Neogymnobatesluteus* (81.77%)	no: different species identification
Fam. Tectocepheidae							
*Tectocepheusalatus* Berlese, 1913	1	BOLD:AEI6504*	ASAP37	mlPTP5	no	*Scutovertexsculptus* (85.44%)	no: different species identification
Fam. Trhypochthoniidae							
*Trhypochthoniusjaponicus* Aoki, 1970	1	BOLD:AEI0244*	ASAP52	mlPTP30	no	*Trhypochthoniustectorum* (82.95%)	no: different species identification
Fam. Xenillidae							
*Xenillusdiscrepanscanariensis* Pérez-Íñigo, 1976	1	BOLD:AEH9009*	ASAP51	mlPTP42	yes/endemic	Arachnida (80.16%)	no: higher taxonomic resolution
*Xenillus* sp1	1	BOLD:AEI9187*	ASAP15	mlPTP50	NA	Sarcoptiformes (81.48%)	NA
*Xenillus* sp2	1	BOLD:AEH9719*	ASAP3	mlPTP41	NA	Scheloribatidae (78.5%)	NA
*Xenillus* sp3	2	BOLD:AEI3741*	ASAP4	mlPTP52	NA	*Parachipteriapunctata* (80.93%)	NA
*Xenillus* sp4	3	BOLD:AEI6505*	ASAP4	mlPTP53	NA	Sarcoptiformes (80.81%)	NA
*Xenillustegeocranus* (Hermann, 1804)	2	BOLD:AEI8992*	ASAP15	mlPTP51	yes/native	Liacaridae (82.13%)	no: higher taxonomic resolution
