# Supplementary figures and images for: Towards a Canary Islands barcode database for soil biodiversity: revealing cryptic and unrecorded mite species diversity within insular soils
Source: Biodivers Data J. 2024 Jan 10;12:e113301. doi: 10.3897/BDJ.12.e113301 (PMC10838043; doi:10.3897/BDJ.12.e113301)

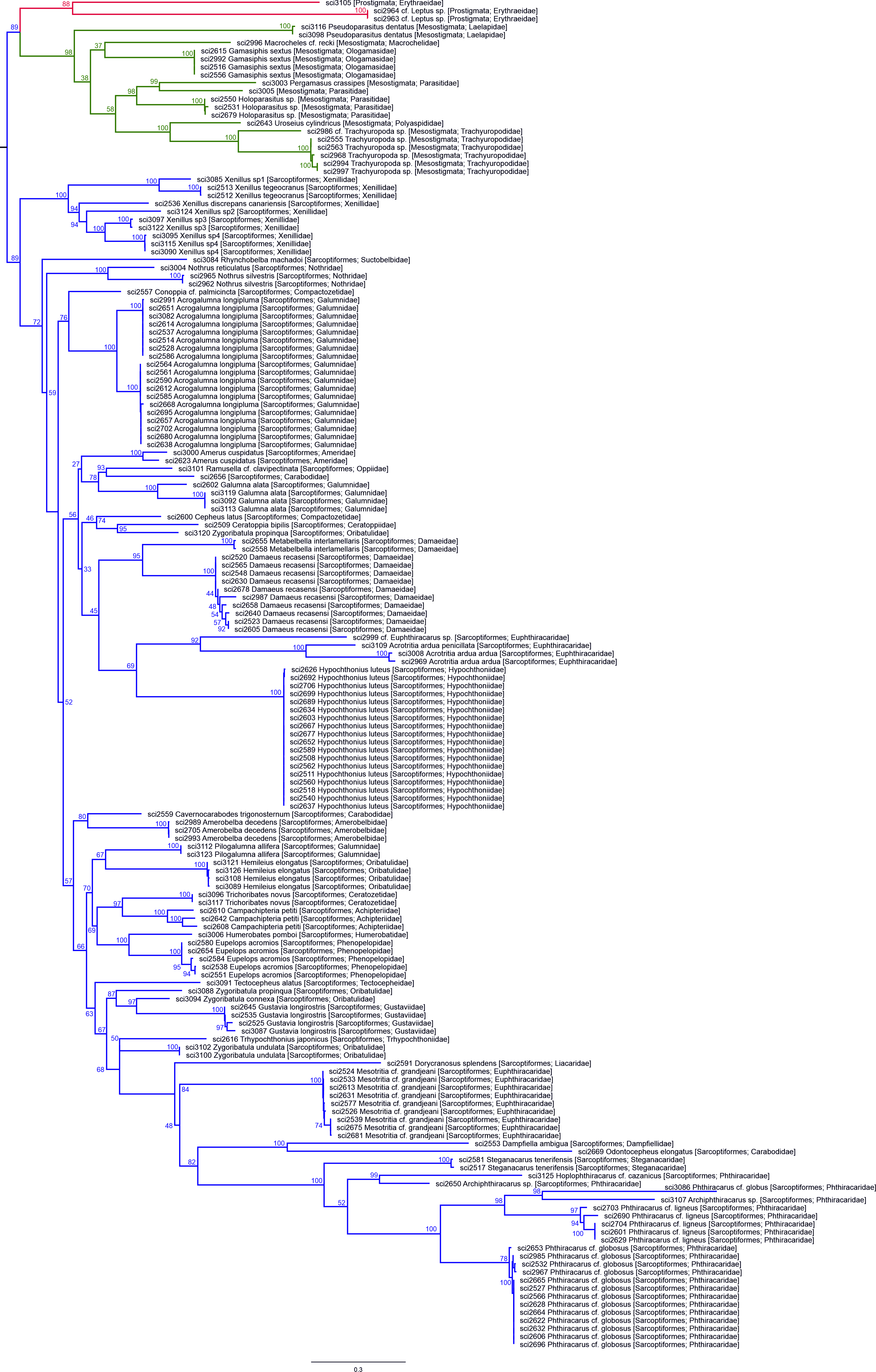

Supplement: Supplementary material 2 — Figure S1 [file bdj-12-e113301-s002.tif]

Figure S2a. *Acrogalumna longipluma* A BOLD tree

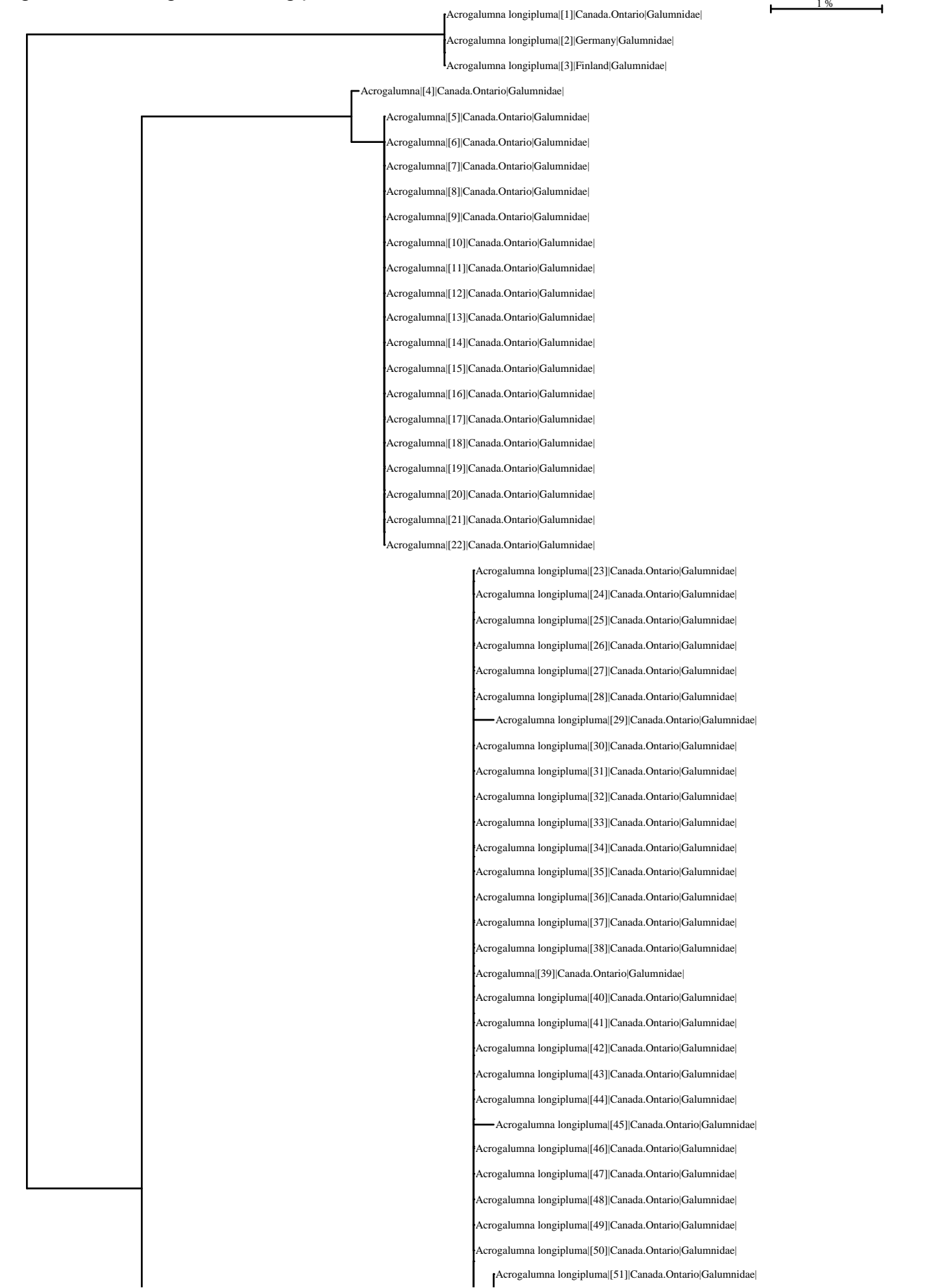

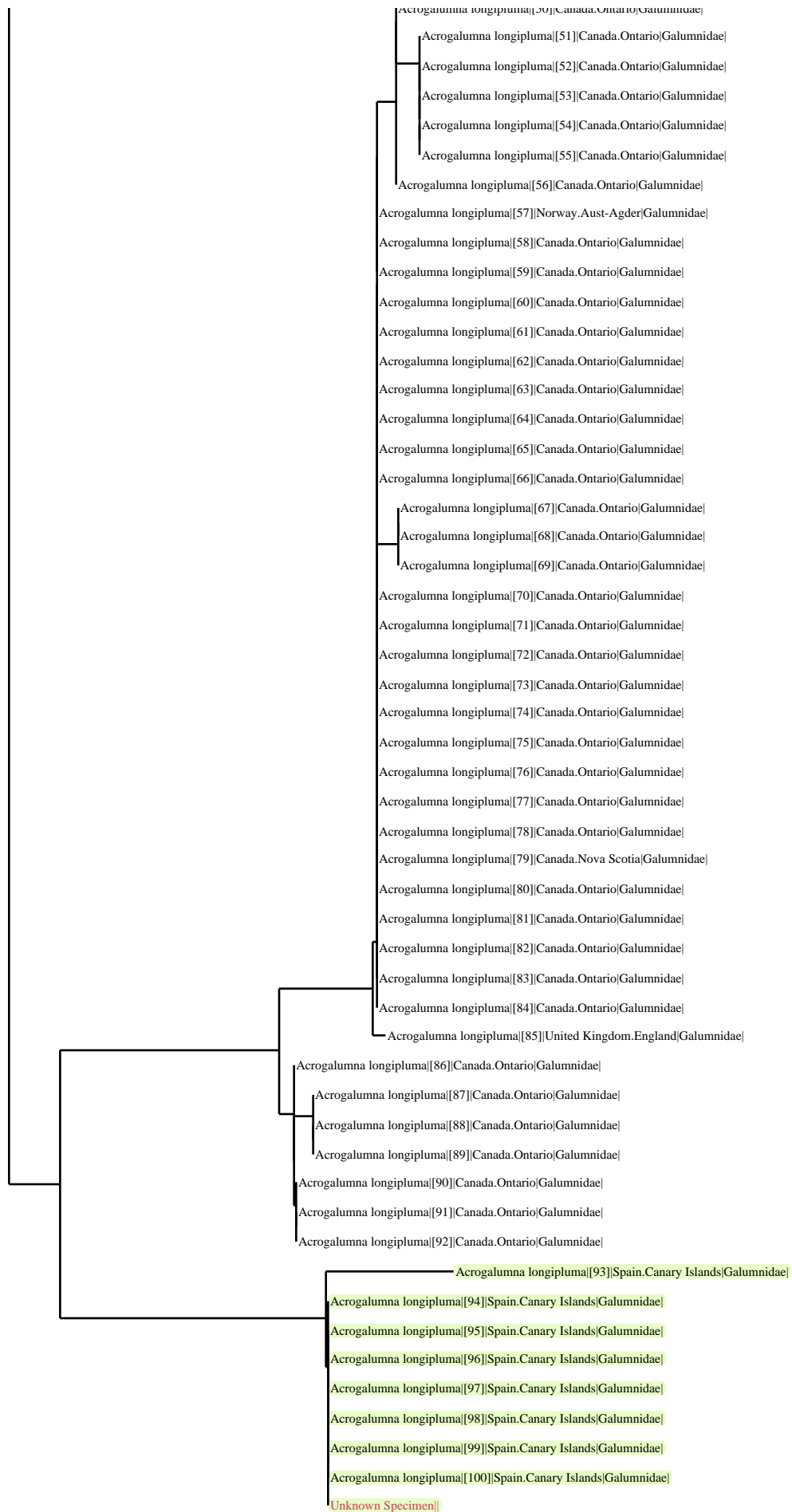

Supplement: Supplementary material 5 — Figure S2a [file bdj-12-e113301-s005.pdf]

Figure S3. *Hypochthonius luteus* BOLD tree

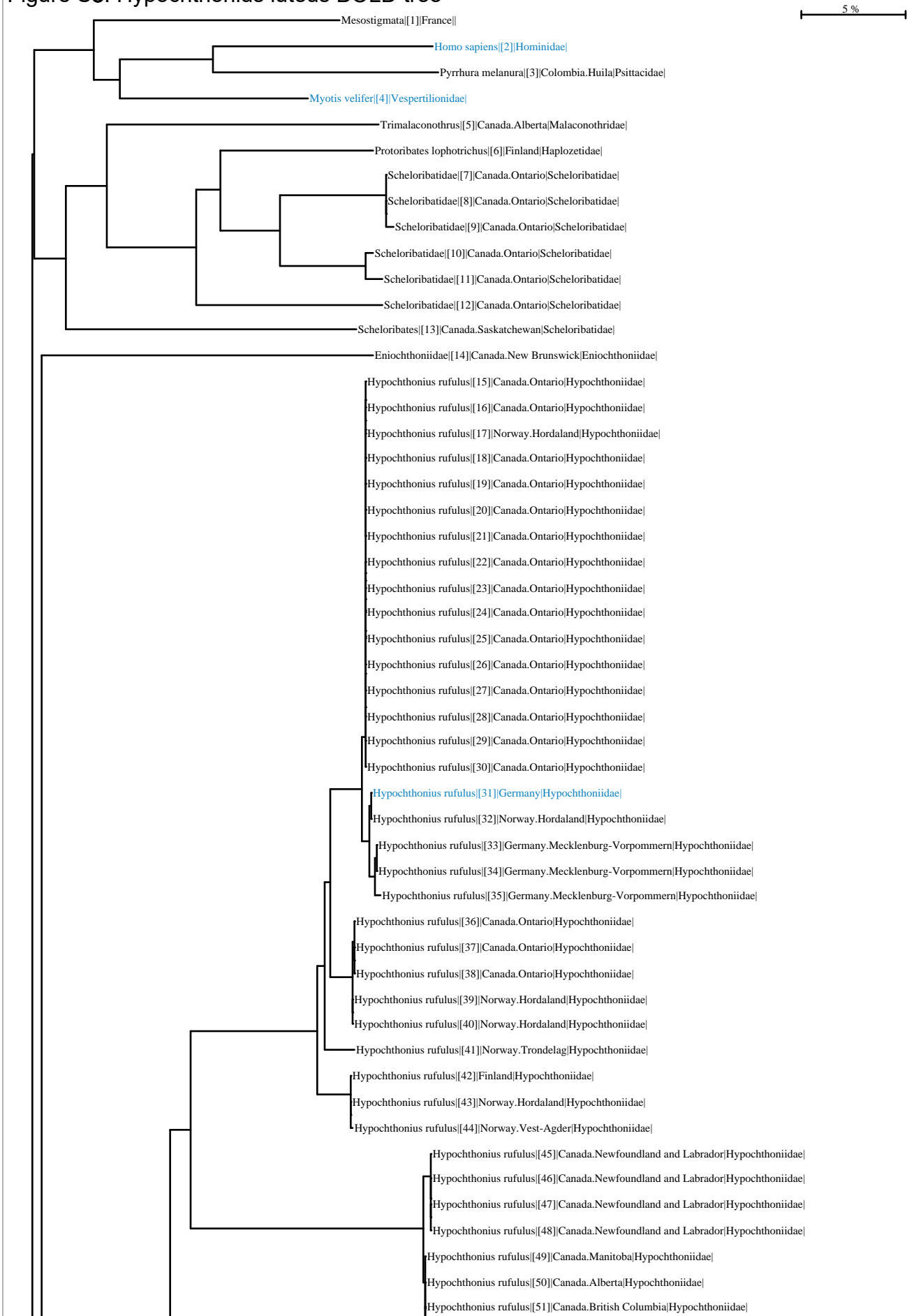

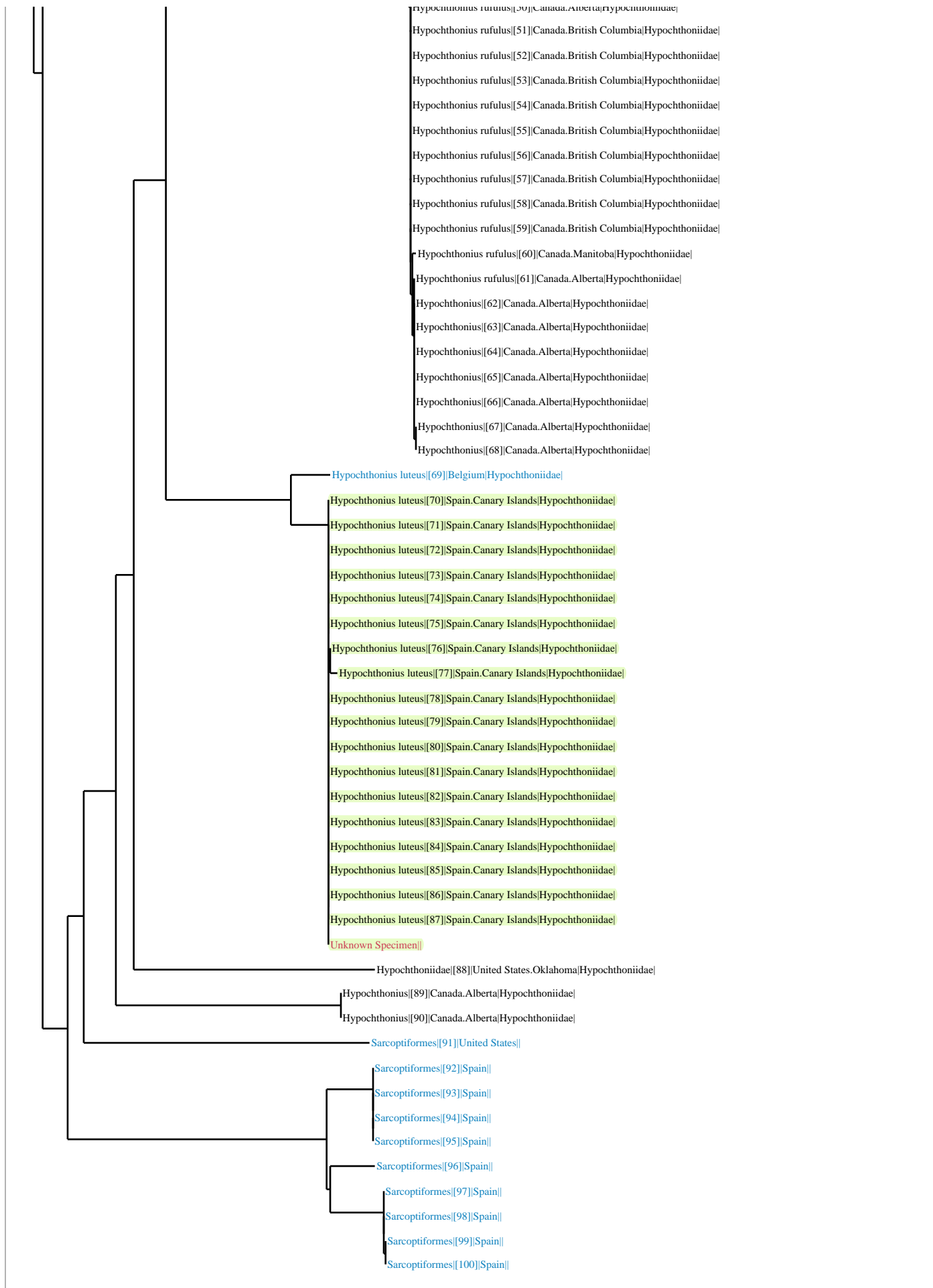

Supplement: Supplementary material 6 — Figure S2b [file bdj-12-e113301-s006.pdf]

Figure S4. *Odontocephus elongatus* BOLD tree

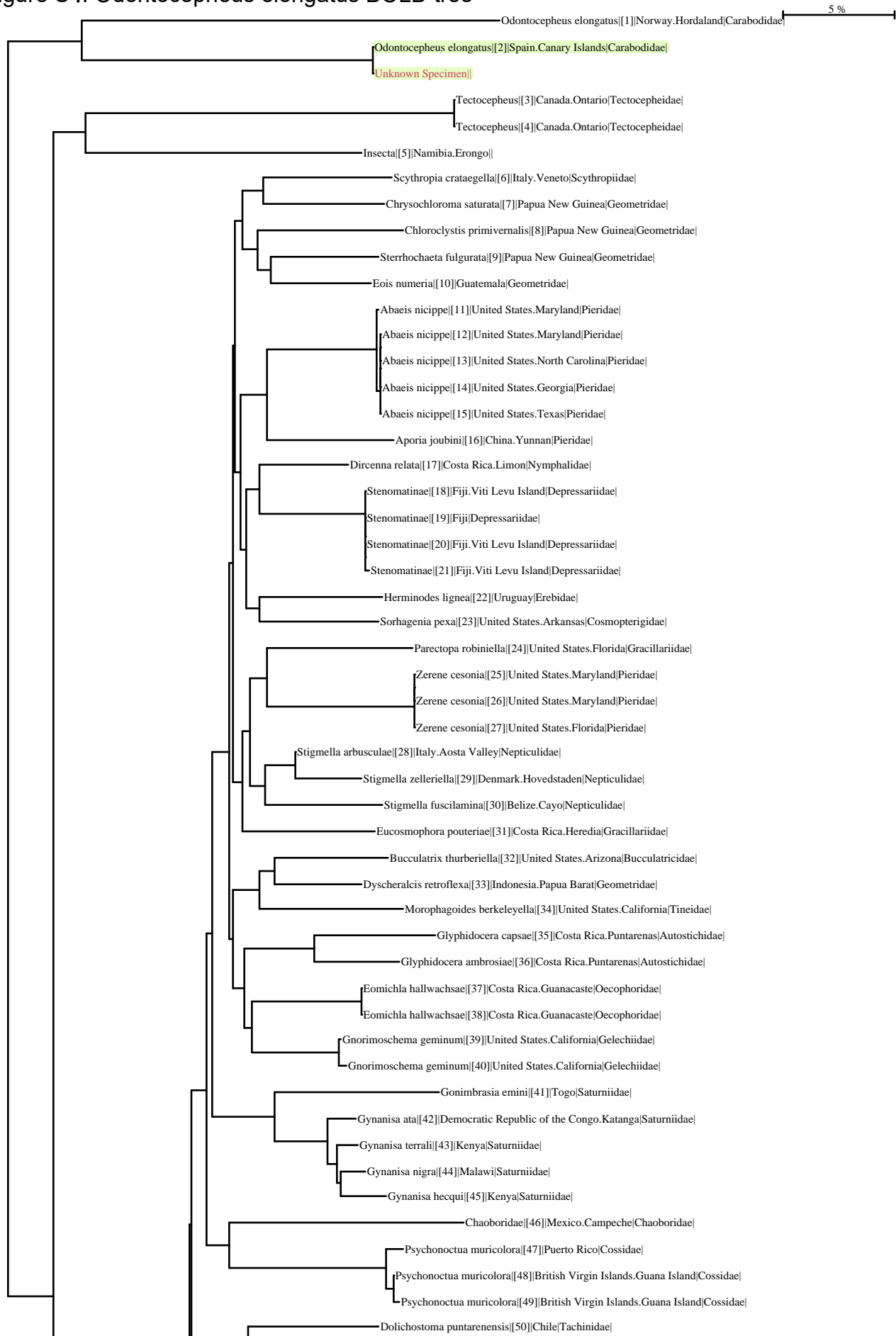

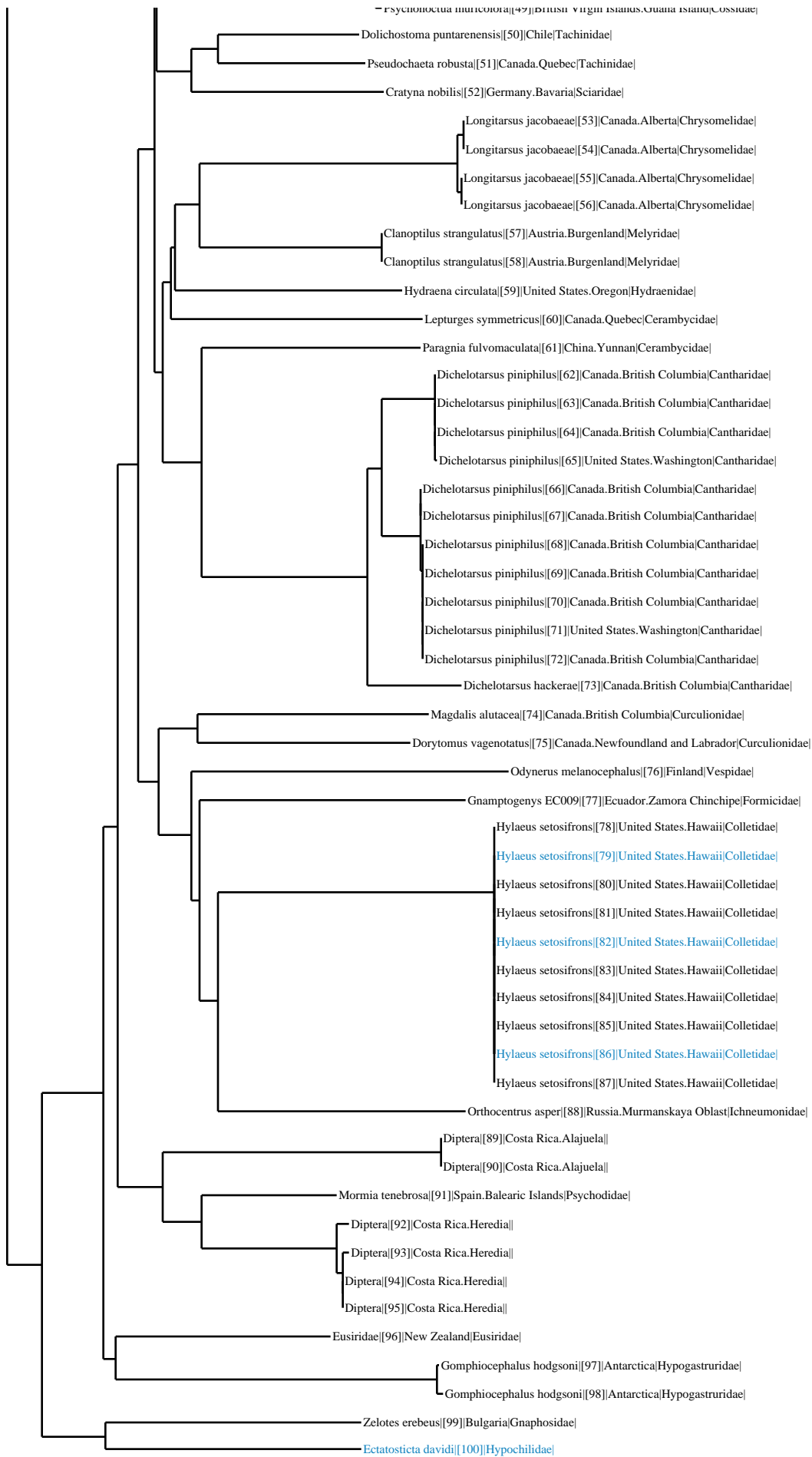

Supplement: Supplementary material 8 — Figure S4 [file bdj-12-e113301-s008.pdf]

Figure S5b. *Acrotrititia penicillata* BOLD tree

2 %

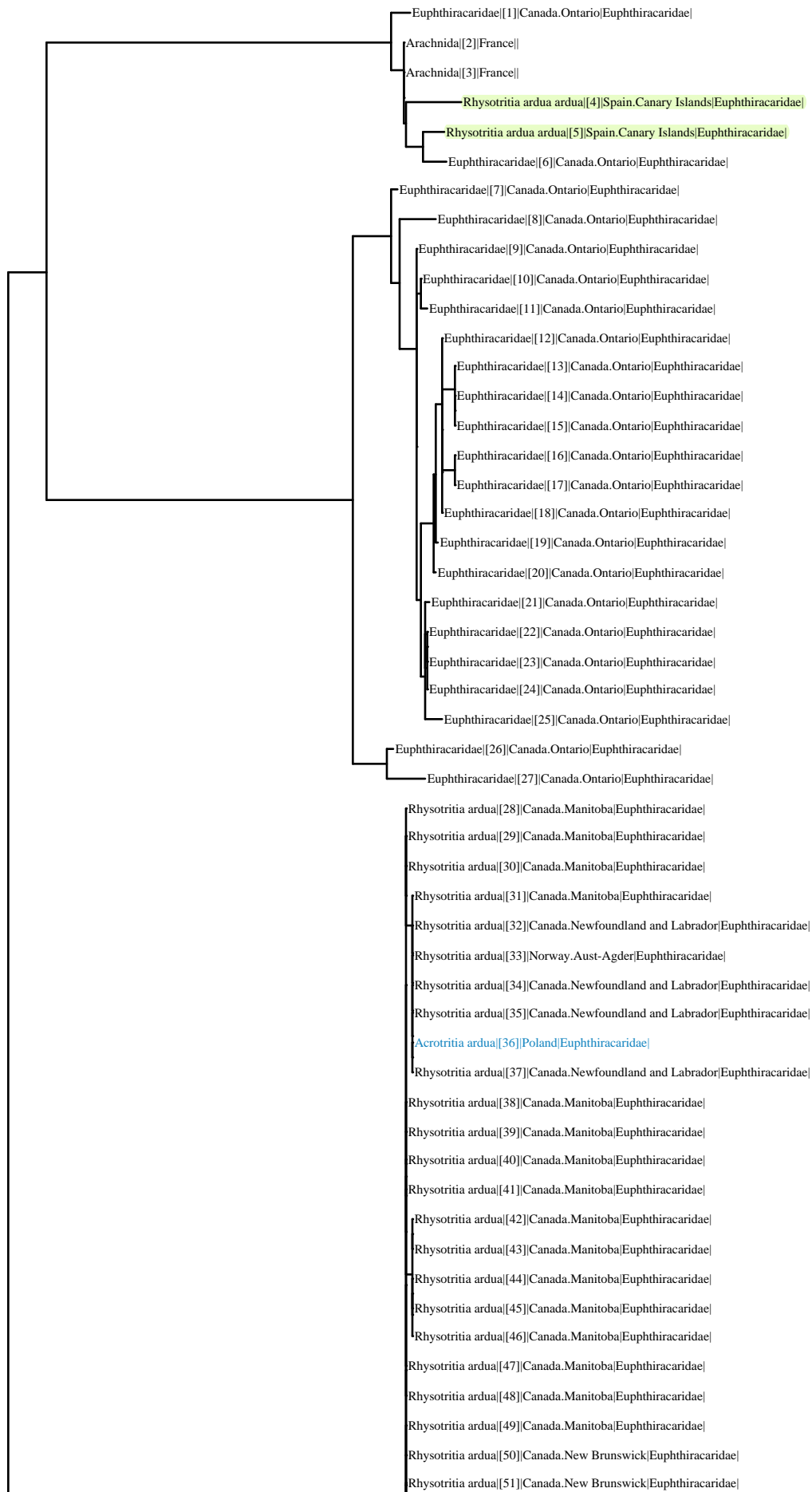

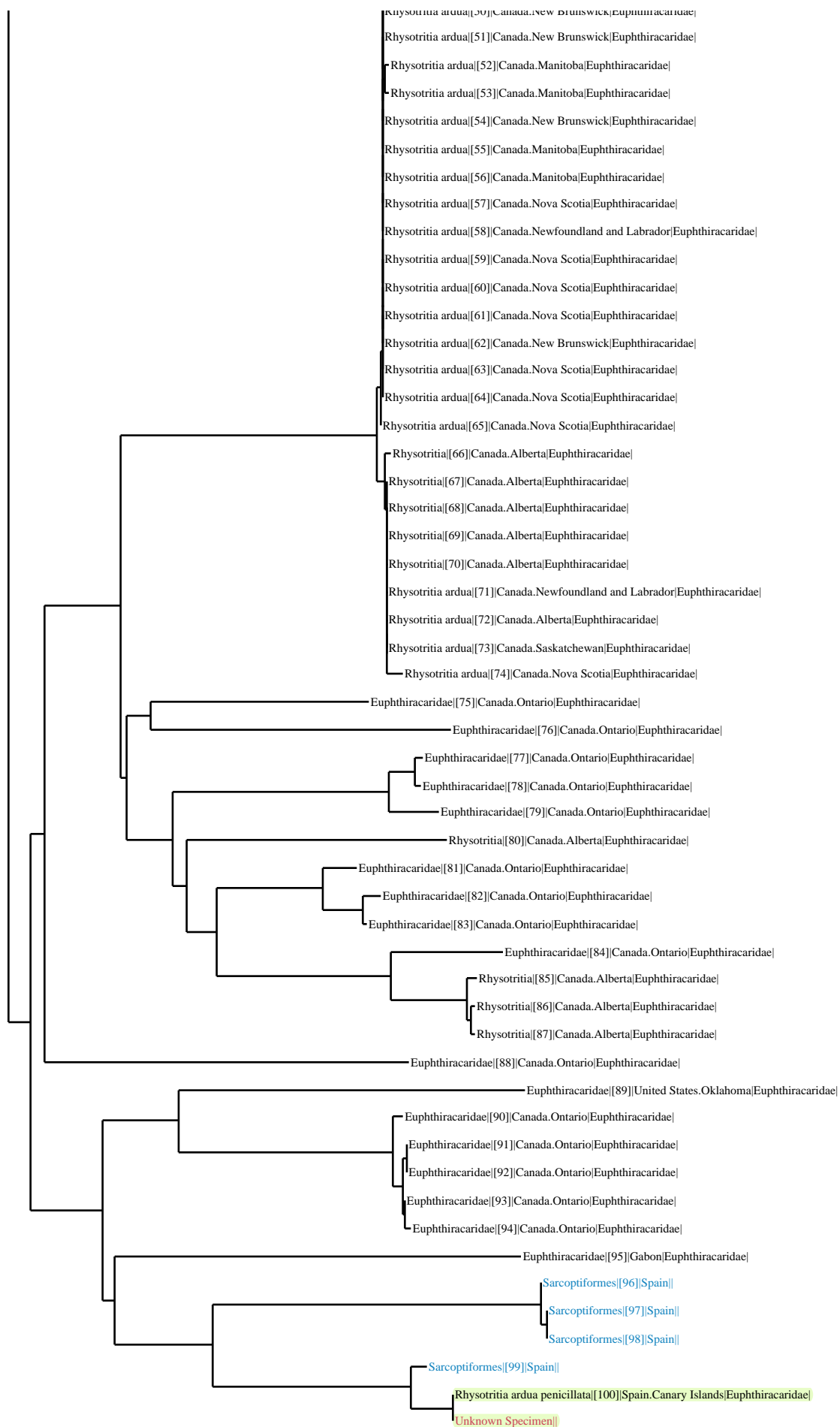

Supplement: Supplementary material 9 — Figure S5a [file bdj-12-e113301-s009.pdf]

Figure S6. *Phthiracarus cf. globosus* BOLD tree

2 %

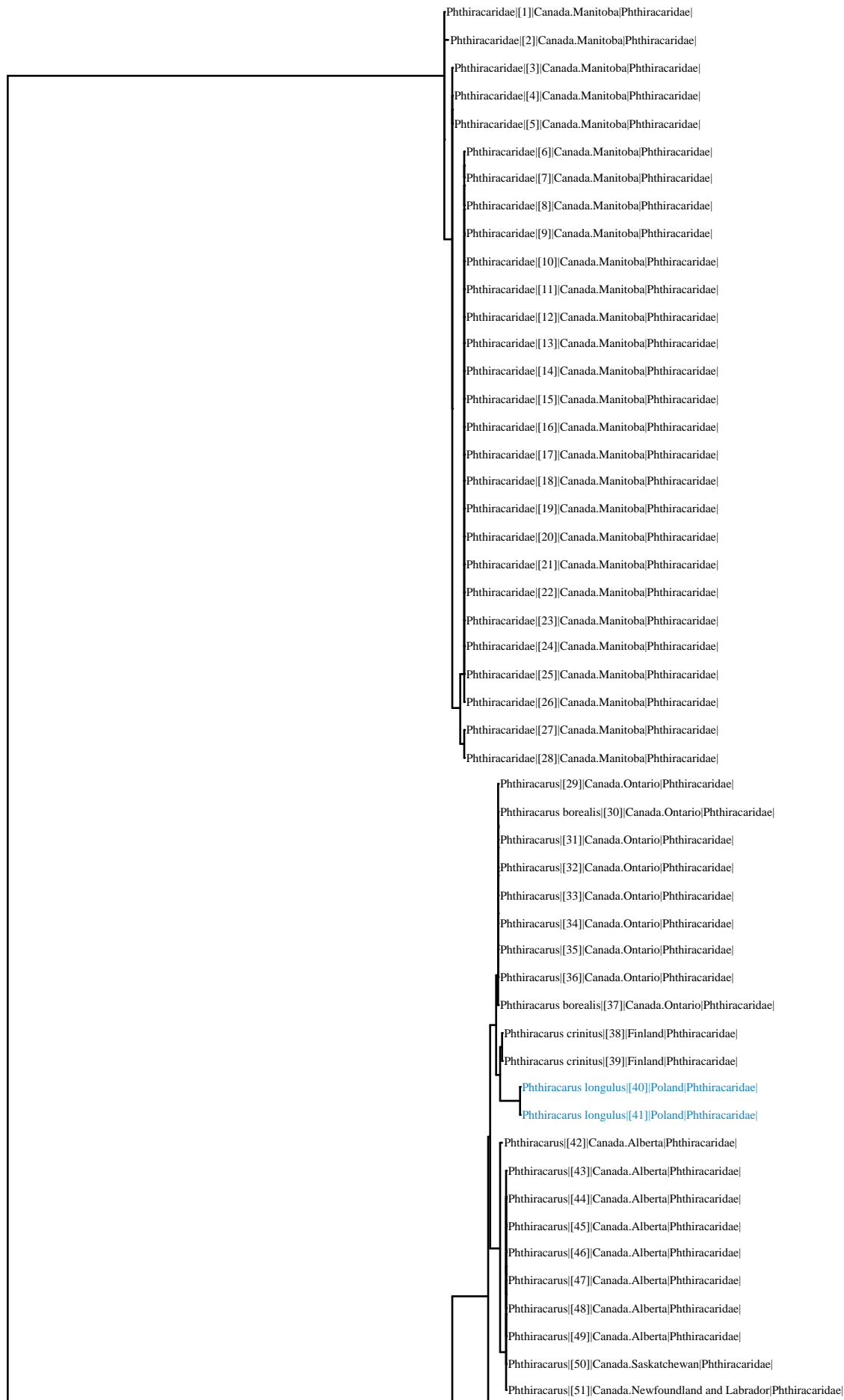

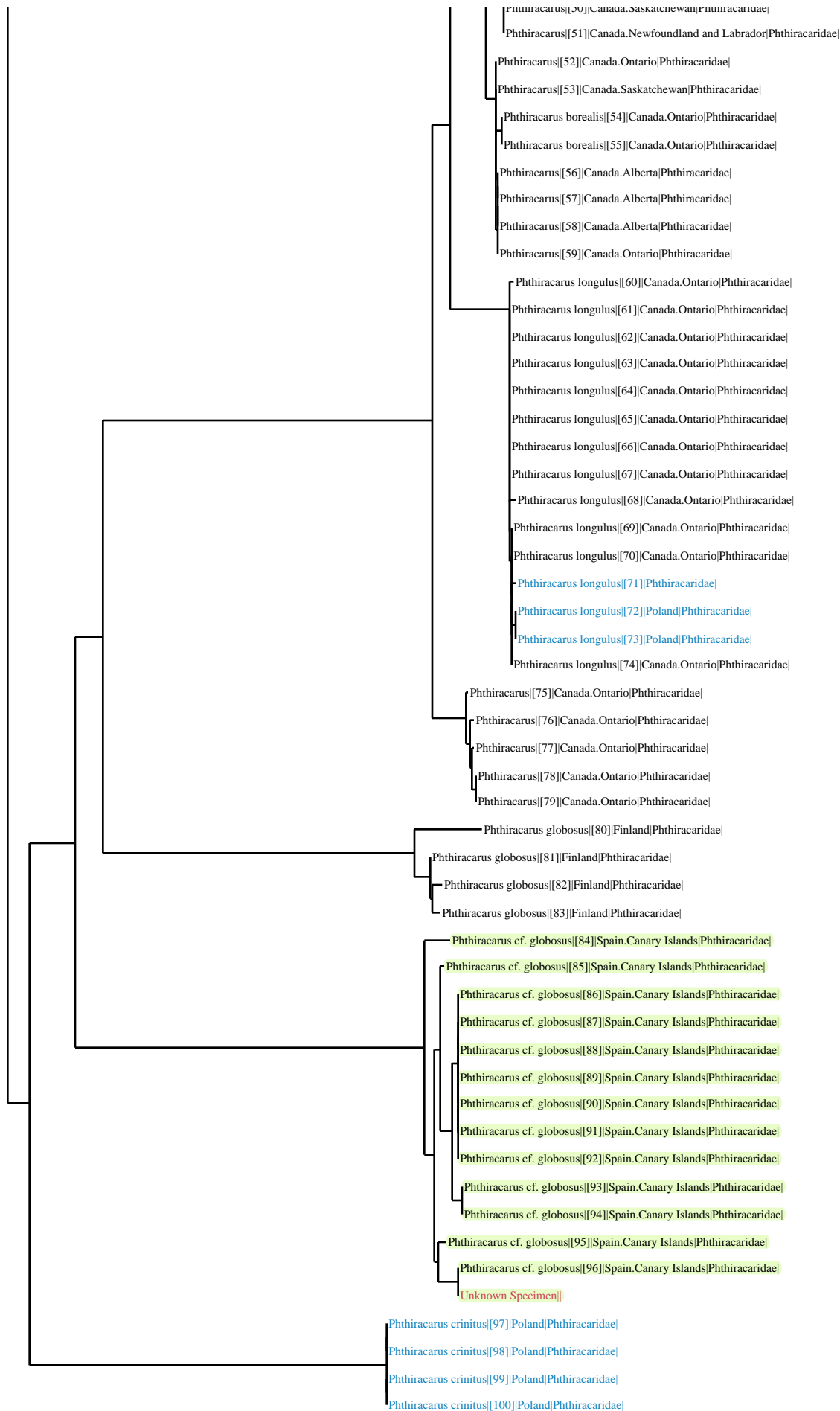

Supplement: Supplementary material 10 — Figure S5b [file bdj-12-e113301-s010.pdf]

Figure S7. *Xenillus tegeocranus* BOLD tree

2 %

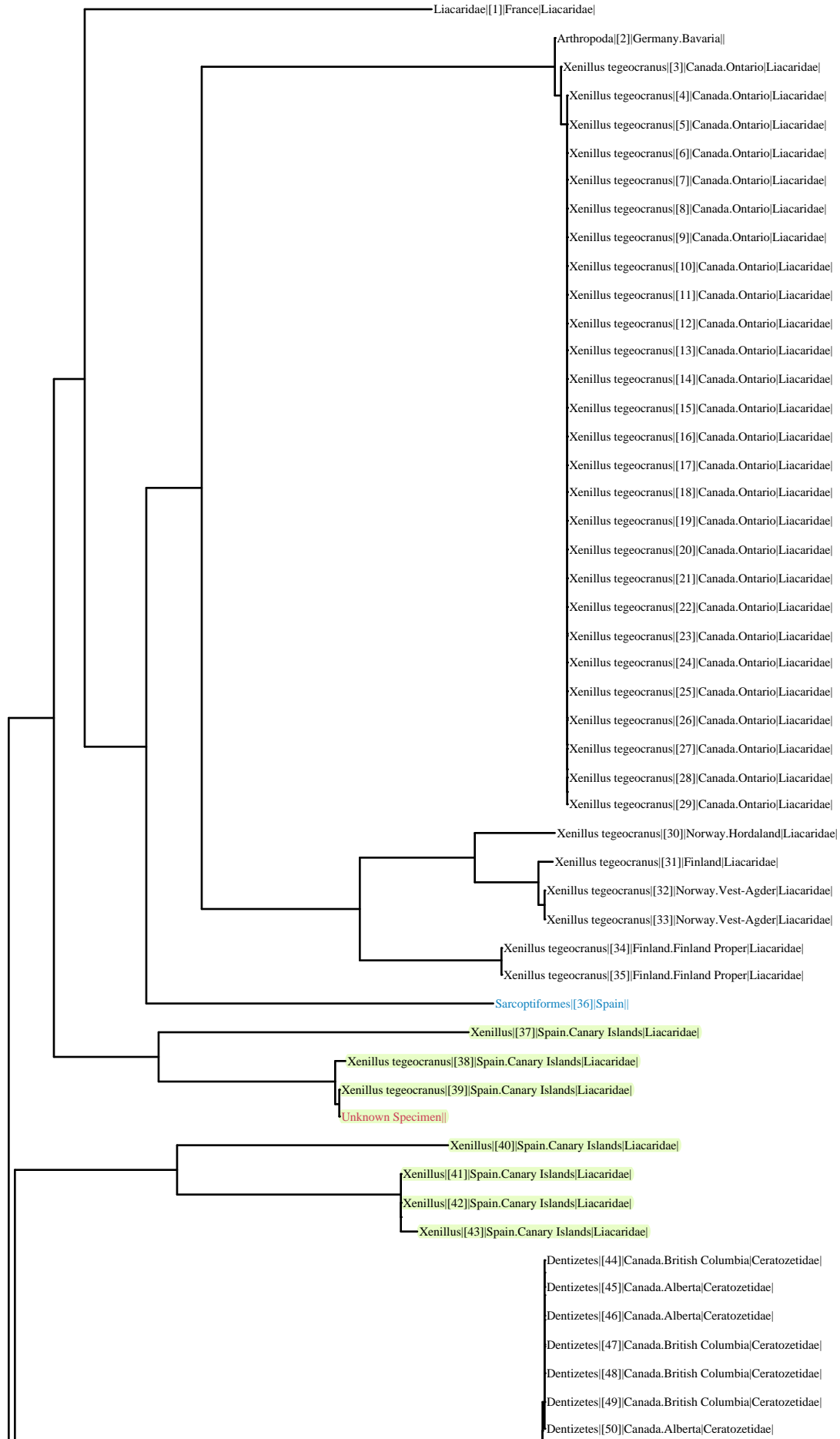

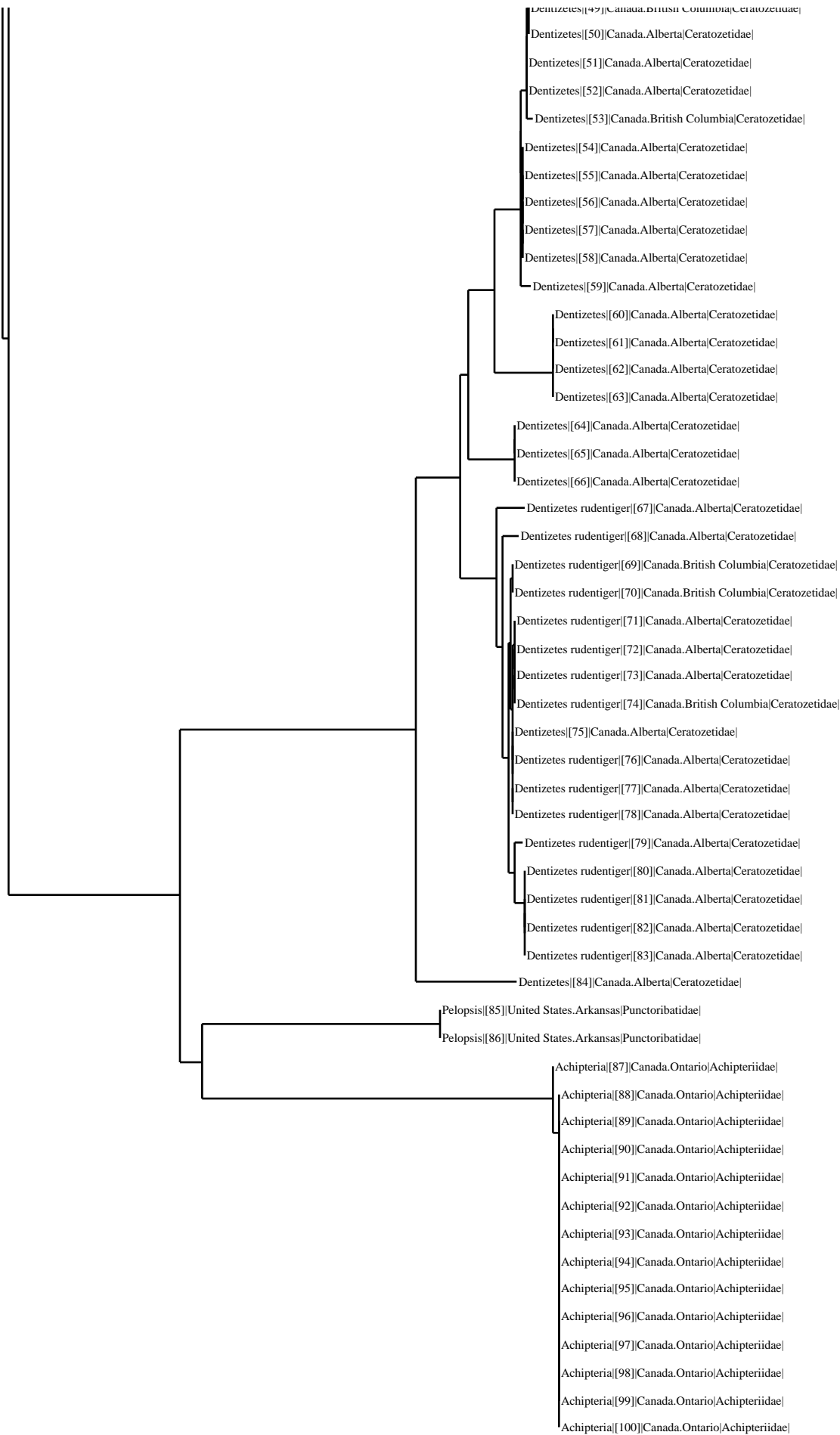

Supplement: Supplementary material 12 — Figure S7 [file bdj-12-e113301-s012.pdf]
